# Exploring the Metabolic Landscape of AML: From Haematopoietic Stem Cells to Myeloblasts and Leukaemic Stem Cells

**DOI:** 10.3389/fonc.2022.807266

**Published:** 2022-02-10

**Authors:** Yashar Mesbahi, Toby N. Trahair, Richard B. Lock, Patrick Connerty

**Affiliations:** ^1^ Children’s Cancer Institute, Lowy Cancer Centre, University of New South Wales (UNSW) Sydney, Kensington, NSW, Australia; ^2^ School of Women’s and Children’s Health, University of New South Wales (UNSW) Sydney, Kensington, NSW, Australia; ^3^ University of New South Wales Centre for Childhood Cancer Research, University of New South Wales (UNSW) Sydney, Kensington, NSW, Australia; ^4^ Kids Cancer Centre, Sydney Children’s Hospital, Randwick, NSW, Australia

**Keywords:** acute myeloid leukaemia, metabolic plasticity, leukaemic stem cells, cancer metabolism, metabolic targeting

## Abstract

Despite intensive chemotherapy regimens, up to 60% of adults with acute myeloid leukaemia (AML) will relapse and eventually succumb to their disease. Recent studies suggest that leukaemic stem cells (LSCs) drive AML relapse by residing in the bone marrow niche and adapting their metabolic profile. Metabolic adaptation and LSC plasticity are novel hallmarks of leukemogenesis that provide important biological processes required for tumour initiation, progression and therapeutic responses. These findings highlight the importance of targeting metabolic pathways in leukaemia biology which might serve as the Achilles’ heel for the treatment of AML relapse. In this review, we highlight the metabolic differences between normal haematopoietic cells, bulk AML cells and LSCs. Specifically, we focus on four major metabolic pathways dysregulated in AML; (i) glycolysis; (ii) mitochondrial metabolism; (iii) amino acid metabolism; and (iv) lipid metabolism. We then outline established and emerging drug interventions that exploit metabolic dependencies of leukaemic cells in the treatment of AML. The metabolic signature of AML cells alters during different biological conditions such as chemotherapy and quiescence. Therefore, targeting the metabolic vulnerabilities of these cells might selectively eradicate them and improve the overall survival of patients with AML.

**Graphical Abstract:**
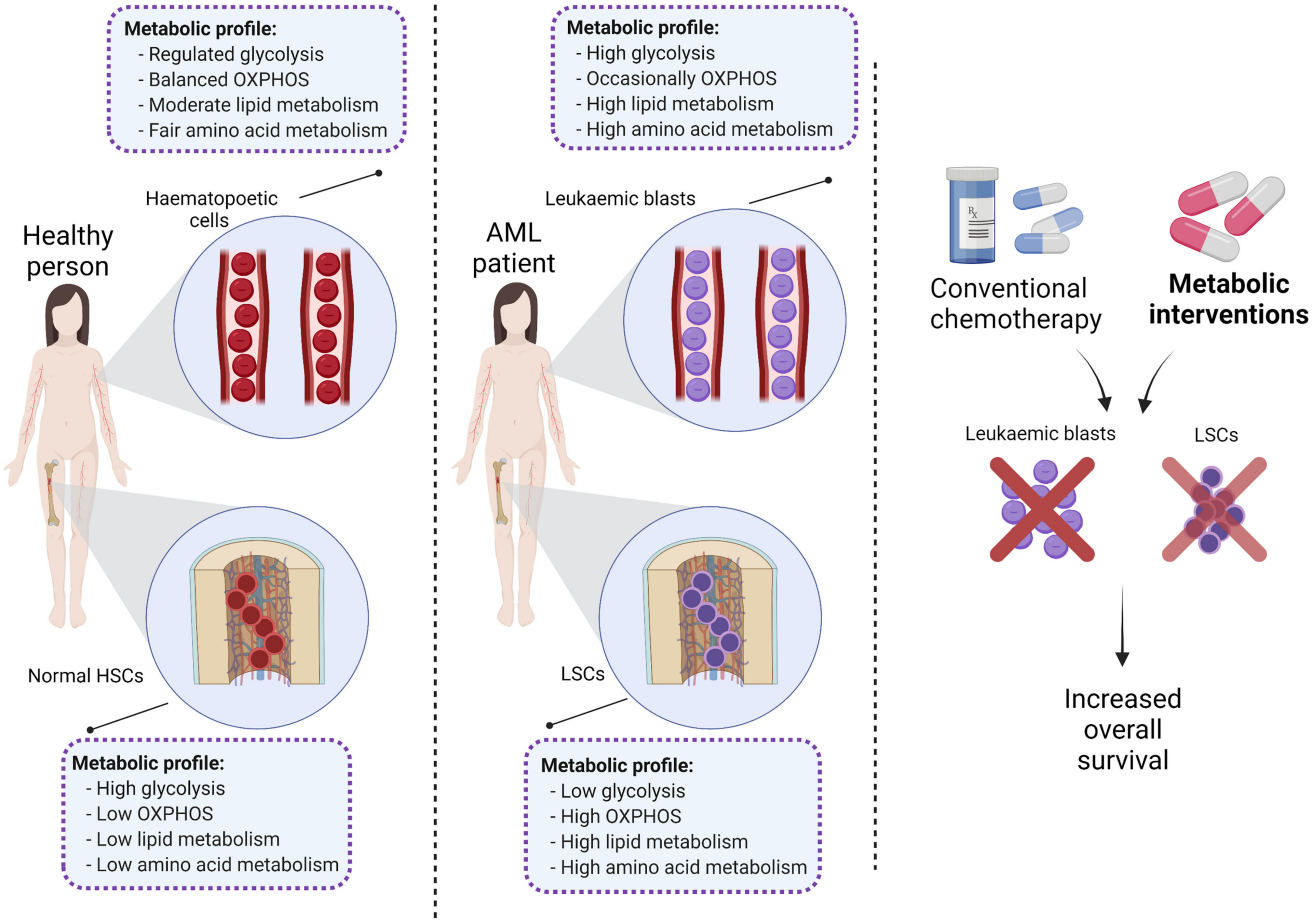


## Introduction

Acute myeloid leukaemia (AML) is the most common acute leukaemia in adults (1, 2). It is characterised by abnormal proliferation of undifferentiated haematopoietic cells called leukaemic blasts (3). The incidence of AML follows an age-dependent pattern, accounting for around 23% of leukaemias in adults (1). Several treatment strategies have been proposed to improve clinical outcomes in patients with AML including allogeneic stem cell transplantation, monoclonal antibodies, and small-molecule inhibitors targeting key leukemogenic drivers. However, combination chemotherapy remains the mainstay of disease treatment (4). Historically, AML treatment has been divided into induction and consolidation phases. The backbone of induction therapy consists of the “7+3” regimen, comprising cytarabine (Ara-C) for 7 days with 3 days of anthracyclines including daunorubicin, doxorubicin or idarubicin (4, 5).

Despite intensive treatment regimens, the median survival rate remains disappointingly low in adults and the majority of patients will eventually succumb to complications of drug treatment or disease relapse. Treatment outcome is significantly better in younger patients with a complete remission rate of ≥80%, however, due to relapse and refractory disease, there is still a 5-year overall survival rate of ~40% (4). Therefore, targeting AML is challenging and requires in-depth knowledge of the underlying cellular and molecular mechanisms which drive the disease.

Metabolism is defined as a series of dynamic processes that allow energy production according to cellular demands. Metabolism is therefore dependent on a cell’s state of proliferation, differentiation, and quiescence (6). For instance, normal cells employ a well-organised network of metabolic programs and a balanced turnover between supply and demand (6). Normal haematopoietic stem cells (HSCs) are characteristically quiescent and adapt their metabolic profiles at a low demand status to maintain their survival and produce multipotent progenitors in the bone marrow (BM) niche (7, 8). In contrast, the bulk AML population is comprised of rapidly proliferating cells that require additional sources of energy for growth and survival (9).

**Figure 1:**
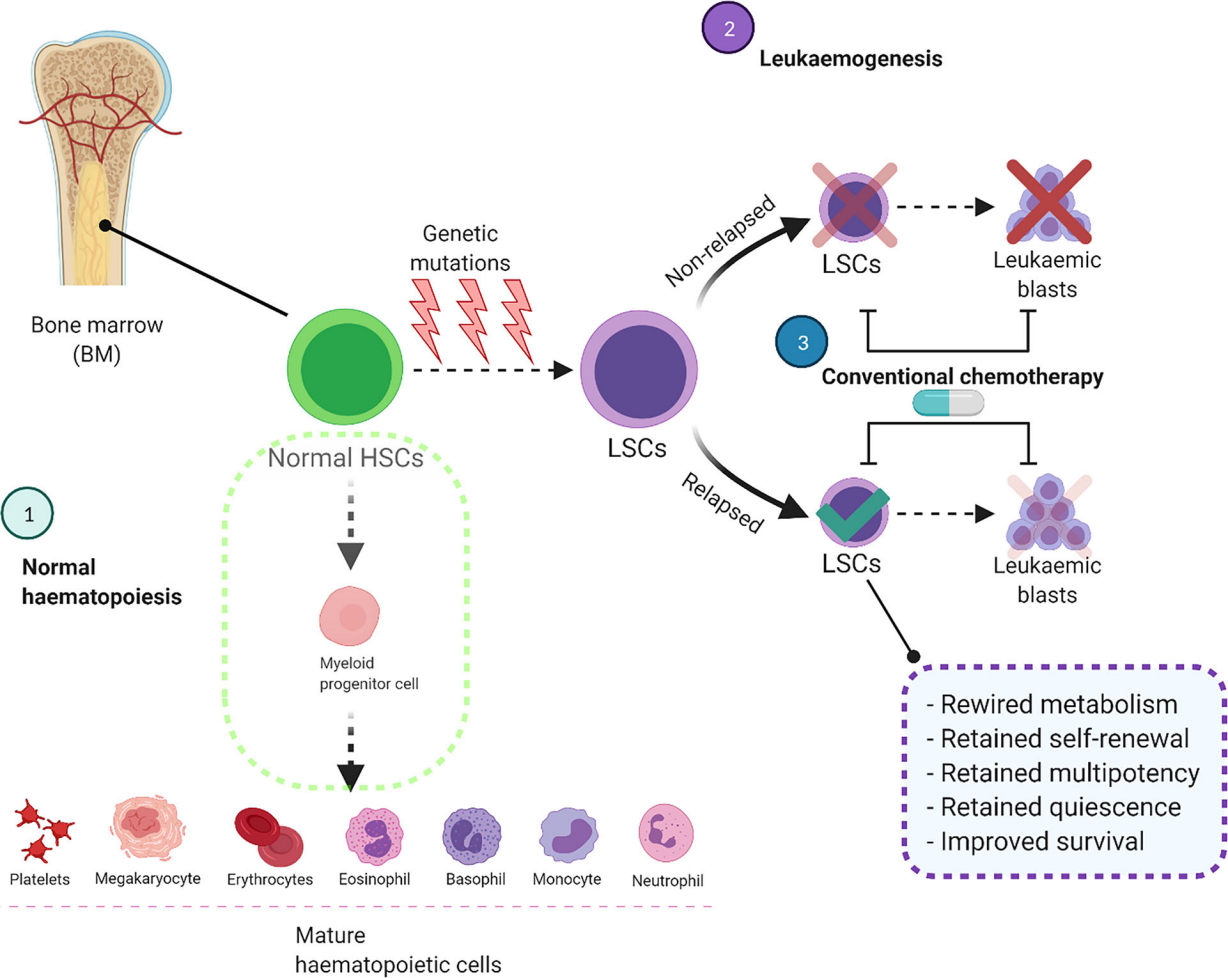
Conventional chemotherapy is ineffective to target and eliminate LSCs, leading to AML relapse. In the normal haematopoietic system, HSCs differentiate into myeloid progenitor cells and eventually produce mature haematopoietic cells. In AML these HSCs acquire genetic mutations which impair the differentiation process and convert them to LSCs. These cells acquire self-renewal ability and produce leukaemic blasts in the bone marrow and other organs. Although the current treatment strategies eliminate leukaemic blasts, they are unable to destroy LSCs completely which will cause AML relapse. BM, Bone marrow; HSCs, Haematopoietic stem cells; LSCs, Leukaemic stem cells.

During normal haematopoiesis, HSCs produce multipotent progenitors that, over multiple cycles of proliferation and differentiation, will produce the entire repertoire of haematopoietic cells (**Figure 1**) (10). However, occasionally these myeloid stem/progenitor cells acquire genetic aberrations, transform into malignant leukaemic stem cells (LSCs), and overproduce immature CD34^+^/CD38^+^ blast cells which do not undergo differentiation (3, 11). As well as metabolic differences (12), HSCs, LSCs and AML blasts are genetically and phenotypically distinct and have high levels of heterogeneity within each cell population (13, 14). Pioneering studies functionally characterised LSCs as a rare subset of the immature CD34^+^/CD38^-^ population which is capable of initiating leukaemia in immunodeficient mice (11, 15). In contrast, more mature CD34^+^/CD38^+^ AML blasts failed to propagate the disease under the same conditions (15). However, as LSCs and normal HSCs share similar CD34^+^/CD38^-^ surface immunophenotype, more research has been done to identify unique membrane markers for LSCs including CD32, CD44, CD47, CD123, TIM3, CD45RA and CD96 (14, 16, 17).

Genome and metabolome heterogeneity is also prevalent within sub-populations of unique haematopoietic cell types. For example, recent studies indicated that within certain immunophenotypic fractions such as CD34^+^/CD38^-^, other factors such as reactive oxygen species content are able to identify which cells are functional LSCs (18, 19). Furthermore, mathematical modelling studies of leukaemogenesis have indicated that mature myeloid cells can often have the same genotype as leukaemic blasts, highlighting that differentiation of leukaemic blasts is not completely blocked in the disease (20). A comprehensive single-cell sequencing study by Bernstein et al. has indicated intra-tumoural heterogeneity of malignant AML cells and uncovered that some undefined phenotypic markers may be expressed by both malignant and normal cells (21). This further reinforces the fact that identification of immunophenotypes might not be powerful enough to detect the origin of leukaemogenesis and investigating other molecular markers such as metabolites and metabolic pathways would help determine disease initiating populations in AML (22).

Like normal HSCs, LSCs reside in the BM niche of AML patients (23) which supports their survival and protects them against chemotherapeutic drug treatment (24–26). In addition, hypoxic conditions in the bone marrow microenvironment (BMME) result in lower reactive oxygen species (ROS) content which not only distinguishes LSCs from AML blasts, but also contributes to maintaining a quiescent cellular status, promotes anaerobic metabolism, and sustains stemness (27, 28); all of which protect LSCs from chemotherapy (29, 30). Unlike quiescent LSCs, circulating AML blasts have higher levels of ROS (18, 31) and upregulate multiple metabolic pathways to supply the required energy for proliferation (19).

Previous studies have extensively focused on the metabolic profile of AML and the rationale for targeting deregulated metabolic pathways in leukaemic blasts (9, 32–35). However, the metabolic landscape of different haematopoietic cells remains unclear and needs further elucidation. Therefore, this review will focus on the differences between the metabolic profiles of normal haematopoietic and AML cells. It will then focus on the metabolic differences between normal HSCs, LSCs, and AML blasts as a means to rationally develop novel treatment approaches to eliminate cells responsible for AML.

## Metabolic Differences Between HSCs, LSCs and Leukaemic Blasts

**Figure 2:**
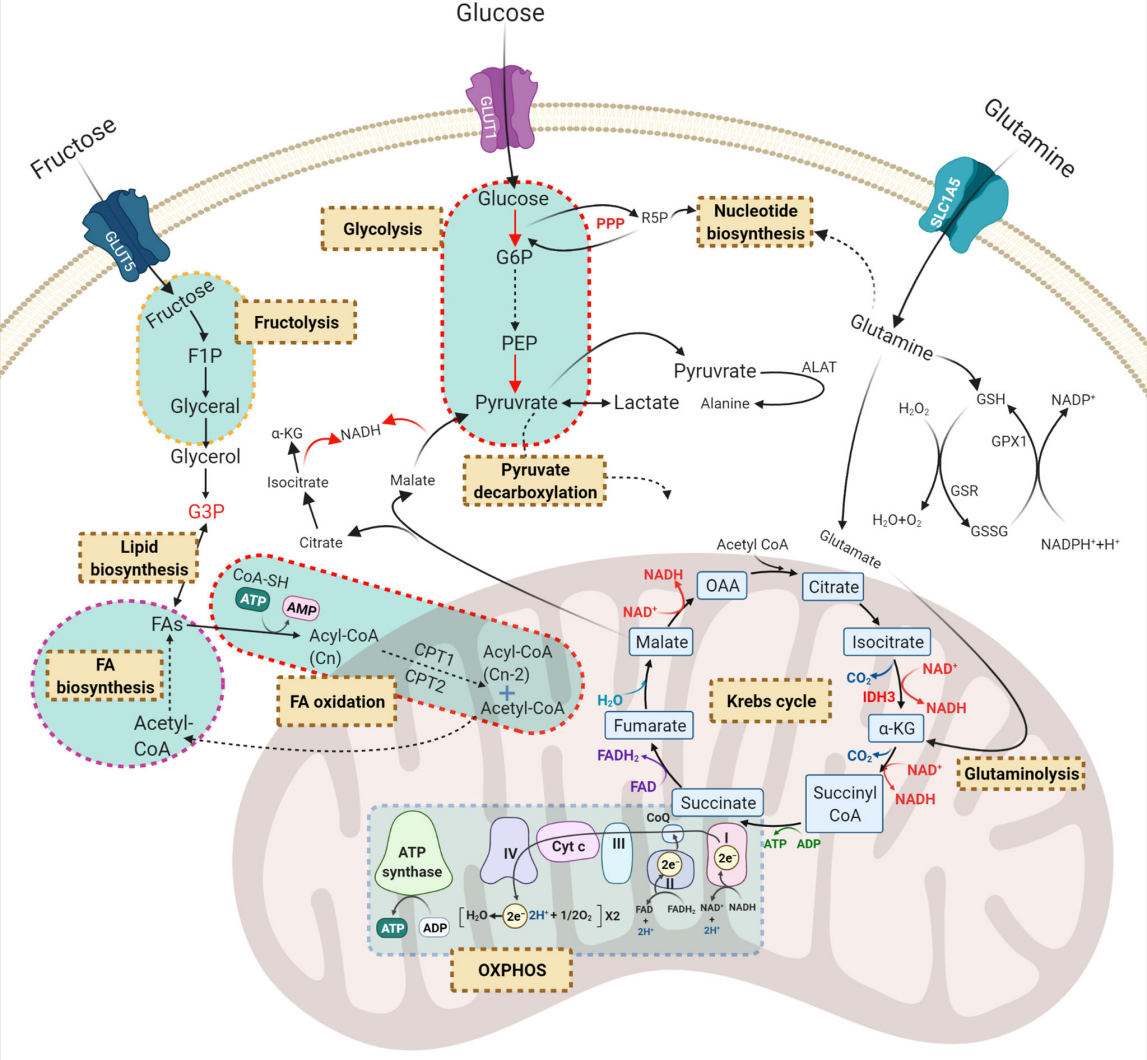
Principal dysregulated metabolic pathways in AML. Carbohydrates and amino acids are two main sources of energy for AML cells which can be used in other metabolic pathways. *Red*, *green*, and *purple* texts are critical compounds in the relevant pathways. *Brown-cream rectangles*, indicating the crucial metabolic processes required for cell survival and proliferation. GLUT, glucose transporter; G6P, glucose-6-phosphate; R5P, ribose-5-phosphate; F1P, fructose-1-phosphate; PEP, phosphoenolpyruvate; G3P, glycerol-3-phosphate; OAA, oxaloacetate; α-KG, α-ketoglutarate; PPP, pentose phosphate pathway; ALAT, alanine transferase; GSH, glutathione; GSR, glutathione-disulfide reductase; GPX1, glutathione peroxidase 1; FA, fatty acid; GLS, glutaminolysis; OXPHOS, oxidative phosphorylation.

Previous reports have indicated that normal HSCs, LSCs and AML blasts have distinct and unique metabolic profiles. Compared to the low energy demands of normal HSCs (36), leukaemic blasts require higher production of ATP to support their increased cellular division (37). Leukaemic blasts upregulate metabolic pathways such as glycolysis (37) and the pentose-phosphate pathway (38) to produce the building blocks of macromolecules including amino acids, nucleotides, fatty acids (FAs) and electron carriers that are necessary for maintaining the leukaemic state (33, 39). In contrast, LSCs depend more on mitochondrial metabolism to maintain their quiescence and self-renewal ability (18, 40). Notable reprogrammed metabolic pathways in AML include glycolysis, oxidative phosphorylation (OXPHOS), amino acid synthesis, and lipid synthesis (**Figure 2**).

## Glucose Metabolism

Nearly a century ago, Dr Otto Warburg discovered that even in the presence of oxygen, tumour cells prefer to ferment glucose to convert pyruvate into lactate rather than allowing it to enter the tri-carboxylic acid (TCA) cycle. This provides cancer cells with a high rate of ATP production to fuel rapid cell division (41). Besides its role in bioenergetics, glucose metabolism involves other pathways including the pentose-phosphate pathway which generates pentose phosphate for ribonucleotide synthesis, serine biosynthesis which generates amino acids and one-carbon metabolism which generates NADPH as the universal electron carrier (42). Therefore, glycolysis not only generates energy but also serves as a platform to produce molecular building blocks for cancer (42).

To initiate glycolysis, AML cells take up glucose by special membrane transporters called GLUTs encoded by the solute carrier family 2A gene. Higher glucose content in both AML cell lines and blasts derived from patient samples correlated with the overexpression of GLUT1 and lactate dehydrogenase leading to drug resistance and tumour cell survival (43). Similarly, higher amounts of pyruvate and lactate were observed in the serum of AML patients at diagnosis compared to healthy controls and were associated with poor survival (37). Pyruvate and lactate are two crucial compounds of glycolysis that are produced in the final glycolytic reactions catalysed by PKM2 and LDHA respectively. Interestingly, deleterious mutations of *PKM2* impaired progenitor cell function without perturbing HSCs, while *LDHA* deletion significantly blocked the function of both HSCs and progenitors during haematopoiesis, demonstrating a key metabolic difference among distinct haematopoietic cells (44).

Glycolysis is controlled through a network of signalling molecules including AMPK and PI3K/Akt/mTOR (45, 46). This network interrelates with all other metabolic pathways such as OXPHOS, the pentose phosphate pathway, and nucleotide biosynthesis which are necessary for normal haematopoiesis. In the case of AML, many pathways are dysregulated, thereby allowing blast cells to proliferate faster and drive leukaemia. For example, it has been shown that loss-of-function mutations of AMPK suppressed leukemogenesis by perturbing the glucose flux *via* downregulation of the GLUT1 transporter (47). Similarly, mTORC1 is highly expressed in LSCs compared to HSCs and inhibition of mTOR complex 1 (mTORC1) was shown to suppress leukemogenesis (38). mTORC1 plays a crucial role in several cellular processes including glycolysis and the pentose phosphate pathway. Moreover, mTORC1 induces glucose addiction and its inhibition enforces AML cells to generate their ATP from OXPHOS instead of glycolysis, which eventually leads to oxidative stress and DNA damage (38). Therefore, although leukaemic blasts prefer glycolytic metabolism to survive and proliferate, they can rewire their metabolic profile and rely on OXPHOS to resist chemotherapy.

mTORC1 is induced by the PI3K/Akt pathway which is constitutively active in 50-80% of AML cases and is associated with decreased overall survival (48). A comprehensive protein array profiling showed that >60% of primary AML cells are characterised by high PI3K/Akt phosphorylation and activity (48). Moreover, the PI3K/Akt axis plays an essential role in normal haematopoiesis and leukemogenesis by regulating glucose uptake and glycolytic flux (49, 50). The PI3K/Akt pathway is essential for the functionality of normal HSCs while its dysregulation depleted the normal HSC cell population and induced myeloproliferative disease and AML in mouse models with constitutive activation of PI3K/Akt (49). Another study by Xu et al. demonstrated that the PI3K/Akt/mTOR pathway is constitutively active in primary AML blasts and is required for their survival while normal HSCs don’t rely on mTOR for long- or short-term survival (51). In the same study, inhibition of the PI3K/Akt/mTOR pathway with rapamycin re-sensitised LSCs to the topoisomerase II inhibitor etoposide without toxicity against normal HSCs, which highlights the role of this pathway in LSC population maintenance (51).

Furthermore, in AML cells harbouring an Fms-like tyrosine kinase-3 internal tandem duplication (*FLT3-ITD*), a common leukaemic mutation that confers a poor prognosis in AML patients, glycolysis is associated with the pentose phosphate pathway to sustain a high flux of glucose for cell survival (52). In a separate study, glucose-6-phosphate dehydrogenase was identified as a crucial regulator of the pentose phosphate pathway and its overexpression correlated with an adverse prognosis (38). Suppression of glucose-6-phosphate dehydrogenase and pharmacological inhibition of FLT3 with lestaurtinib induced a significant anti-leukaemic effect in AML cells with *FLT3-ITD* and was identified as a potential therapeutic strategy (53). In contrast to AML blasts, the role of the pentose-phosphate pathway has not been thoroughly investigated in normal HSCs and malignant LSCs and further *in vivo* and *in vitro* studies are needed in these cells.

An important regulator of carbohydrate consumption and glycolysis is the BMME. HSCs and ROS-low LSCs are both dependent on the BMME to survive. HSCs supply their energy mainly through glycolysis, whereas ROS-low LSCs achieve energy metabolism through mitochondrial respiration (19, 54). In contrast, rapidly dividing leukaemic blasts can take up much higher amounts of glucose and fructose in the peripheral blood. In line with this, suppressed fructose uptake has been found to reduce leukemogenesis and intensify the cytotoxicity of Ara-C in fast-proliferative cells in the BMME. This suggests that rapidly proliferating AML cells in the BMME rely on carbohydrate metabolism to confer resistance to Ara-C (55).

**Figure 3:**
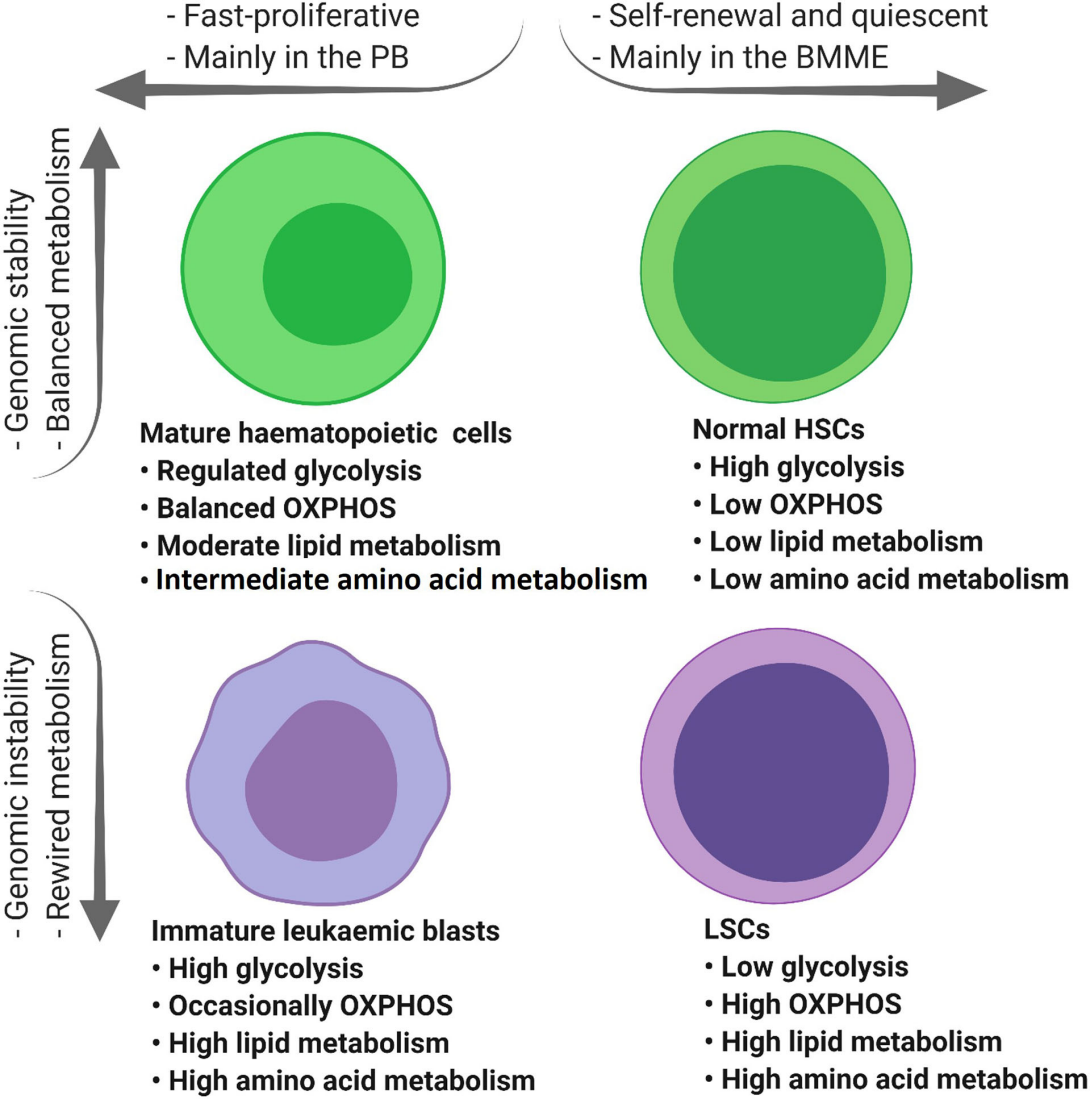
The tumour microenvironment alters the metabolic profile of different types of haematopoietic cells. Mature haematopoietic cells and normal HSCs maintain their genome integrity and therefore regulate their metabolic profile in a balanced manner (green cells). In contrast, immature leukaemic blasts and LSCs with an unstable genome display dysregulated metabolic profiles (purple cells). PB, peripheral blood; BMME, bone marrow microenvironment; OXPHOS, oxidative phosphorylation; TCA, tricarboxylic acid cycle; ETC, electron transfer chain; HSCs, haematopoietic stem cells; FAO, fatty acid oxidation; AAs, amino acids; LSCs, leukaemic stem cells.

Collectively, compared to LSCs, normal HSCs and rapidly proliferating AML blasts have higher glucose content and glycolytic activity (**Figure 3**) (37, 56) which highlights the role of glucose metabolism in leukemogenesis.

## Mitochondrial Metabolism

Mitochondria are classically considered as the powerhouse of cancer cells, where multiple metabolic pathways that feed on carbohydrates, amino acids and fatty acids converge into the TCA cycle (57). In addition to bioenergetics functions, the mitochondria environment supports and coordinates main metabolic processes including the TCA cycle, glutaminolysis, OXPHOS and fatty acid oxidation (FAO) (58). Some of these mitochondrial pathways are significantly altered in AML cells and are vital for AML cell survival and function.

### The TCA or Krebs Cycle

The TCA cycle serves as a central metabolic hub that collects high-energy electron carriers, such as NADH and FADH_2_, from glycolysis and delivers them to the electron transport chain (ETC) in the mitochondria (59). Normal HSCs have a well-organised metabolic profile in which the TCA cycle is triggered by the glycolysis end-stage product, pyruvate (**Figure 2**). However, AML blasts with a dysregulated metabolic signature disconnect the association of glycolysis and the TCA cycle by enforcing cells to convert pyruvate to lactate. This uncoupling assists AML cells to utilise other carbon sources such as glutamate to fulfil their energy demands. Therefore, targeting glutamine metabolism could potentially serve as an effective therapy for AML (60).

Cancer cells convert glutamate to α-ketoglutarate (α-KG) by glutamate dehydrogenase as an auxiliary reaction to replenish TCA intermediates (61). In line with this notion, CRISPR/Cas9 knockout screens indicate that AML cell lines enrich for glutamine transporters (SLC1A5, SLC38A1, and SLC38A2) and glutaminase (GLS) to supply α-KG for the TCA cycle, which has been shown to protect them against the anti-proliferative effects of the BCL-2 inhibitor ABT-199 (venetoclax) (62). These results frame a classic concept that cancer cells shunt carbon from glutamine into citrate which then can be fed into the TCA cycle (63). TCA cycle activity is also upregulated in ROS-low LSCs but not in ROS-high AML blasts. ROS-low LSCs showed significant ^13^C_16_ palmitate uptake which was incorporated into the TCA cycle intermediates citrate and malate (18). In contrast to LSCs and rapidly dividing AML cells, metabolomic profiling of quiescent long-term HSCs showed low levels of TCA metabolites (2-oxoglutarate, acetyl-CoA and succinyl-CoA) which are regulated by HIF-1α; a transcription factor responsive to cellular hypoxia. HSCs harbouring HIF-1α loss-of-function mutations exhibited decreased dependence on glycolysis and impaired quiescence (64).

In the TCA cycle, α-KG is produced from isocitrate which is catalysed by isocitrate dehydrogenase (IDH). However, it has been shown that somatic mutations in the active site of IDH1/2 in AML cells lead to the overproduction of the oncometabolite 2-D-hydroxyglutarate, which blocks differentiation and contributes to AML progression (65). Moreover, IDH2^R140Q^ mutations *in vivo* drive aberrant self-renewal activity and block differentiation in normal HSCs. Furthermore, co-mutant *in vivo* models of IDH2^R140Q^ and FLT3-ITD induced acute leukaemia and were essential for leukaemia maintenance (66). Mutations of IDH1/2 have been shown to promote self-renewal and suppress differentiation of normal HSCs, leading to the clonal expansion of stem/progenitor cells, known as pre-LSCs (67). These mutations persisted in the pre-LSC population in AML patients with long-term complete remission following induction therapy (68). In addition, IDH1 mutations have been shown to be conserved during disease evolution. Matched paired analysis of AML patients revealed identical IDH1 mutations in diagnosis versus relapse samples demonstrating the conservation of IDH1 mutations in LSCs which drive disease relapse (69). In a similar study, Shlush et al. examined the existence of a pre-leukaemic cell population in AML patients with IDH1/2 mutations and indicated that 2 out of 6 samples acquired IDH2 mutations in some progenitor and mature populations (70).

### Oxidative Phosphorylation

It was originally assumed that mitochondrial respiration was impaired in cancer cells as they mainly rely on glycolysis for survival (41). However, it is now understood that tumour cells utilise oxygen in OXPHOS to generate high levels of ATP by transferring electrons to the TCA cycle for cell survival (71). In addition to providing energy, OXPHOS also regulates mitochondrial membrane permeabilisation, controlling the balance between apoptosis and proliferation and playing an important role in redox biology (71).

Normal HSCs maintain their quiescent state by reducing their mitochondrial respiration rate and relying on glycolysis (72). In line with this, Škrtić et al. have shown that AML blasts have higher mitochondrial biogenesis and basal oxygen consumption compared to normal HSCs (73). However, it has also been shown that quiescent HSCs and not AML blasts can maximise their energy production with higher glycolytic activity and electron flux, known as the spare reserve capacity while coping with oxidative stress (46, 72). This implies that normal HSCs can compensate for energy loss by upregulating glycolysis while AML blasts are vulnerable to mitochondrial oxidative stress.

Similar to quiescent HSCs, LSCs also have characteristically low levels of oxygen consumption and OXPHOS, but they still rely on this metabolic pathway for their survival (19). A recent study by Cole et al. found that mitochondrial ATP-dependent Clp protease (ClpP) is overexpressed in the CD34^+^ CD38^−^ LSC population of primary AML samples (74). Therefore, increased ClpP expression might serve as a biomarker of elevated OXPHOS in AML patients. In line with this, metabolomics and gene expression analyses by Jones et al. indicated that LSC-enriched subsets are metabolically dormant populations and, unlike HSCs, preferably depend on amino acid metabolism for OXPHOS-related energy production (18).

Mitochondrial metabolism, the TCA cycle and OXPHOS are critical pathways to produce ATP molecules in LSCs and, under certain conditions, AML blasts. Thus, targeting OXPHOS alone or in combination with other metabolic pathways could be an attractive therapeutic strategy to re-sensitise LSCs and AML blasts to conventional chemotherapeutic agents and improve AML patient outcomes.

## Amino Acid Metabolism

The 20 standard amino acids are required for a diverse set of cellular processes crucial for tumour cell proliferation, including the biosynthesis of proteins, nucleotides, lipids, and glutathione (GSH) (75). In addition to their role in metabolism, amino acids are fundamental in mediating epigenetic regulation and post-transcriptional modification (75).

Among all amino acids, glutamine is the most important for AML survival and proliferation. Along with glucose, glutamine plays a critical role in tumour progression not only to provide α-KG for the TCA cycle but also for the importation of leucine into AML cells, leading to the activation of mTORC1-mediated protein synthesis (60). Glutamine is imported into the AML cells by the SLC1A5 transporter where it is converted into glutamate and α-KG in a process known as glutaminolysis (76). Compared to normal HSCs, leukaemic blasts import relatively more glutamine for their survival and growth (**Figure 3**). In an *in vivo* study, normal HSCs from healthy donors were resistant to apoptosis induction following glutamine removal. In contrast, depletion of glutamine by shRNA knockdown of SLC1A5, induced apoptosis in eight primary AML samples (77), suggesting a reliance on glutamine metabolism in leukaemic blasts compared to normal HSCs. Furthermore, low-glutamine containing cells showed a significant reduction in their oxygen consumption rate compared to high-glutamine cells, suggesting that OXPHOS inhibition is caused by glutamine deprivation (60, 78).

ROS-low LSCs derived from AML patients at diagnosis mainly rely on amino acid metabolism to fuel OXPHOS for survival (18). In contrast, LSCs from relapsed AML escaped amino acid loss by enhancing FA metabolism; mainly by inducing palmitate uptake. Palmitate can be metabolised to TCA cycle intermediates to provide NADH and FADH_2_ for OXPHOS (18). Therefore, to target and eliminate ROS-low LSCs derived from patients with relapsed AML, it is necessary to inhibit subsidiary pathways that provide oxidative-related energy such as the inhibition of fatty acid metabolism in the mitochondria (79). Likewise, a comprehensive proteomic analysis of leukaemic cells revealed a high level of branched-chain amino acid transaminase 1 (BCAT1) in CD34^+^CD38^-^ LSCs compared to the non-LSC AML population. BCAT1 is a negative regulator of α-KG which is a crucial intermediate of the TCA cycle. Besides its role in the TCA cycle, α-KG acts as a co-factor for DNA demethylases such as the Egl-9 family hypoxia-inducible factor 1 and the ten-eleven translocation family. Therefore, the CD34^+^CD38^-^ LSC population with increased levels of BCAT1 shows a hyper-methylated phenotype similar to cases with mutant IDH1/2, in which DNA demethylases are blocked by the oncometabolite D-2-hydroxyglutarate (80). This hypermethylation causes the inhibition of key tumour suppressor genes which lead to LSC development. Accordingly, knockdown of BCAT1 in leukaemia cells caused αKG accumulation leading to demethylases protein degradation and abrogated leukaemia-initiating potential (80).

Aside from glutamine metabolism, AML cells rely on other amino acids for survival such as arginine. Arginine is crucial to provide amine groups and aspartate for the production of nucleotides. Unlike normal HSCs, AML cells with arginosuccinate synthetase-1 mutations are unable to generate arginine from citrulline and aspartate and are dependent on importing extracellular arginine (81). Arginine catabolism is a key player in blocking the immune response to antigens on AML blasts and therefore measurement of plasma arginine could be an important addition to immunotherapy of AML patients (82). Overall, amino acid content and metabolism are more prominent in LSCs compared to AML blasts and present an opportunity to selectively target the LSC population (18, 19, 83).

## Lipid Metabolism

After glucose and glutamine, lipids are the third source of fuel for cancer cells proliferation and survival (84). Lipids are highly complex biomolecules that not only function as an energy source but also provide building blocks for functional fatty acids that are required for cell membrane biogenesis and signalling pathways regulation (85).

Lipid metabolism is often deregulated in AML blasts compared to normal haematopoietic cells (**Figure 3**) implying the potential of targeting lipid synthesis for therapeutic benefit in patients with AML (86, 87). Comprehensive lipidomic profiling of plasma from AML patients at diagnosis and healthy blood donors showed an increase in certain free fatty acids such as arachidonic acid and depletion of total fatty acids and cholesterol which was probably driven by enhanced FAO (86). FAO itself feeds the TCA cycle with acetyl-CoA intermediate, leading to citrate increment which is the starting point of *de novo* fatty acid synthesis (88).

FA synthase (FASN) is a multi-enzyme protein that catalyses FA synthesis. It is required for AML cell proliferation and was shown to be low in normal HSCs derived from healthy donors and highly overexpressed in AML patients. Inhibition of FASN with RNAi or epigallocatechin-3-gallate accelerated granulocytic differentiation in acute promyelocytic leukaemia cells and re-sensitised them to ATRA treatment (89). This process was suggested to be mediated through lysosomal biogenesis and autophagy (89).

Fatty acid oxidation (FAO) is another deregulated pathway in AML, playing an important role in promoting leukaemic cell survival and quiescence (90). FAO generates acetyl-CoA from the oxidation of FAs which is the reverse pathway of FA synthesis (**Figure 2**). In addition to FAO, glycolysis and amino acid catabolism also provide more acetyl-CoA molecules which then will be fed into the TCA cycle to produce ATP (91). Therefore, understanding the molecular mechanisms of one pathway would shed light on others that can be exploited therapeutically. Several enzymes and transporters involved in the process of FAO, including carnitine palmitoyltransferase 1A and carnitine transporter CT2, are overexpressed in AML cells compared to normal HSCs (32, 92, 93). Quiescent HSCs maintain FAO rates at a basal level to preserve their dormancy (94). HSC fate depends on whether they undergo symmetric or asymmetric cell division when they leave quiescence (95). The asymmetric division generates two daughter cells; one will acquire self-renewal ability and remain quiescent and the other will differentiate and enter the circulatory system. However, the symmetric division will generate two daughter cells only capable of undergoing cell proliferation and differentiation (95). FAO metabolism supports asymmetric division and thus preserves the self-renewal ability of HSCs (94).

Similar to HSCs, FAO is important for LSC maintenance and plays a key role in venetoclax resistance (79). Transcriptional profiling of resistant ROS-low LSCs with RAS pathways mutations showed enrichment in FAO and increased CD36 expression (79). CD36 is an important fatty acid transporter that facilitates lipid uptake in the CD36^+^ LSC population. These CD36^+^CD34^+^ cells are significantly enriched in gonadal adipose tissues of the BM which protects them from destructive effects of venetoclax and allows them to utilise FAO required for their maintenance (87). In line with this, it has been shown that the interaction of AML blasts and BM adipocyte cells induces lipolysis, releases FAs from adipocytes to AML and supports AML cell survival, and proliferation *in vivo* (96).

In addition to FAs and related metabolic pathways, sphingolipids also play a critical role in leukemogenesis by regulating the balance between cell proliferation and cell death (97, 98). The formation and functionality of sphingolipids rely on oncogenic proteins including sphingosine kinases and acid ceramidases in AML cells. Sphingosine-1-phosphate is a bioactive lipid that is generated by sphingosine kinase 1 and regulates AML cell survival and death in a constitutively active manner (99). A recent study has demonstrated that S1PR3, a receptor for sphingosine-1-phosphate, is upregulated in AML blasts and CD34^+^CD38^-^ LSCs compared to normal HSCs. S1PR3 regulates myeloid differentiation and activates inflammatory pathways in AML blasts and CD34^+^CD38^-^ LSCs. S1PR3 activation in primitive AML samples promoted LSC differentiation leading to the elimination of these cells (100).

Based on these observations, the metabolism of HSCs, LSCs and AML blasts each rely on distinct pathways for energy production. Therefore, targeting metabolic pathways with selective inhibitors could be a promising strategy to target specific cellular populations in AML.

## The Metabolic Profile of LSCs Drives Drug Resistance

AML is a clonal disorder of haematopoiesis in which normal HSCs or multipotent progenitors acquire genetic mutations that result in dysregulated self-renewal ability (13, 101). These cells are known as “pre-leukaemic HSCs”, representing the evolutionary ancestors of leukaemia (101). In normal haematopoiesis, HSCs eventually differentiate into mature blood cells, but specific genetic mutations interrupt differentiation resulting in LSCs with acquired clonogenic and leukaemia initiating potential (3, 13, 101, 102). These LSCs produce AML blasts and the accumulation of blast cells in the BM defines the disease (**Figure 1**).

The LSC population represent a minor fraction of the disease which is resistant to chemotherapy (103, 104) and are generated by evolutionary processes before the initiation of treatment (104). Following treatment and subsequent remission in patients, relapse of the disease is driven by this drug-resistant CD34^+^CD38^-^ LSC fraction which has unlimited self-renewal capabilities (102). Current treatment regimens are effective against the majority of rapidly dividing bulk AML cells but less successful at eliminating the LSC population due to homing in the BMME which supports their quiescence and self-renewal capacity (**Figure 1**) (105, 106). The BMME is critical for the maintenance and retention of quiescent LSCs and provides crosstalk between LSCs and stromal cells, which significantly influences leukaemia initiation, progression, and response to therapy (106). Furthermore, the gene expression profiles of HSCs and LSCs are profoundly influenced by the BMME cellular architecture and these cells express a dynamic and heterogenous molecular landscape compared to leukaemic blasts (23).

A recent study by van Gils et al. comprehensively categorised six major processes involved in therapy resistance of LSCs; altered epigenetic pathways, cellular plasticity, the tumour microenvironment, integrated stress responses, cellular signalling and metabolic dysregulation (107). Among these processes, the role of metabolic dysregulation in chemotherapy resistance of LSCs has gained favourable attention (24, 108). As a well-known example, the upregulation of ABC transporters in LSCs allows these cells to pump small molecule inhibitors and cytotoxic drugs out of the cell (109). Moreover, much like HSCs, LSCs rarely divide and are maintained in a state of long-term dormancy called quiescence with a low level of oxidative stress and ROS contents (15, 19). AML cells with low ROS levels, defined as ROS-low LSCs, decrease their ATP or oxygen requirements and mainly rely on low energy metabolic pathways to survive and promote leukemogenesis (19). This is a major reason that conventional chemotherapeutics such as Ara-C are unable to eliminate LSCs, as these drugs are more effective against rapidly dividing AML blasts (**Figure 1**) (110). It should be noted that studies have identified a fraction of blast cells with retained mitochondrial activity and a high proliferation rate are less sensitive against Ara-C, implying that different types of haematopoietic cells exhibit varied metabolic profiles which can be exploited for targeting (111).

## Metabolic Targeting in AML

Metabolic targeting is considered a promising therapeutic strategy that is gaining ground in various human cancers including AML. Previous studies have emphasised that metabolic pathways are complex in AML cells and therefore in-depth investigations are required to further shed light on the molecular mechanism of these pathways. Extensive research has been conducted to identify and develop new drugs targeting metabolism in AML. These drugs mainly target glucose metabolism, mitochondrial metabolism, amino acid, and lipid metabolism. Although metabolic interventions have been effective at eliminating leukaemic cells, more comprehensive preclinical studies are needed to increase treatment outcomes and confirm that these drug modulations do not target normal haematopoietic cells. **Table 1** lists information on common metabolic inhibitors that have been tested against AML either in preclinical *in vitro* and *in vivo* studies or clinical trials.

**Table 1:** Modulators of metabolic pathways, preclinical studies, and clinical trials in AML.

Compound	Metabolic target or process	Metabolic pathway	Reference	Study type	Findings
2-DG	Glucose	Glycolysis	(37, 112, 113)	*In vitro* & *in vivo*	Decreased AML cell proliferation, sensitivity to Ara-C
A2-32-01	ATP-dependent Clp protease	OXPHOS	(74)	*In vitro* & *in vivo*	Antileukaemic effects in PDXs & cell lines
Brequinar sodium	DHODH	Nucleotides & OXPHOS	(114, 115)	*In vitro, in vivo* & phase I/II	Reduced leukaemic burden, improved survival & induced differentiation
Rapamycin, 2-DG & 6-AN	mTORC1, glucose & G6PD	Glycolysis & PPP	(38)	Phase I/II	Reduced AML cell viability
Venetoclax	Mitochondrial antiapoptotic BCL-2	OXPHOS & pyrimidine biosynthesis	(18, 19, 116–119)	Phase I/II/III	Selective elimination of LSCs & reduced relapse
Tigecycline	Cox-1 & Cox-2	Mitochondrial protein translation	(73, 120)	*In vitro, in vivo* & Phase I	Antileukaemic activity in PDXs
ddC	mtDNA polymerase	mtDNA replication	(121)	*In vitro* & *in vivo*	Selective elimination of LSCs & induced tumour regression
Enasidenib AG-221	IDH2 mutant enzyme	2-HG production	(122–124)	Phase I/III	Reduced relapse & increased overall survival
ADI-PEG 20	Arginine	Amino acid metabolism	(81)	*In vitro* & *in vivo*	Induced sensitivity to Ara-C & apoptosis
BCT-100	Arginine	Amino acid metabolism	NCT03455140	Phase I/II	Well tolerated without toxicity
L-asparaginase & high-dose Ara-C	Asparagine glutamine availability	Amino acid metabolism	(125–127)	Phase I/II	Increased median survival in relapsed patients
CB-839	Glutaminase	Glutaminolysis	(78, 128)	Phase I	Inhibited AML growth in PDXs & prolonged survival in patients
Avocatin B	CPT1a	Fatty acid oxidation	(129)	*In vitro*	Induced apoptosis
CPI613	PDH	Fatty acid synthesis	(130)	*In vitro* & *in vivo*	Increased sensitivity to doxorubicin
Etomoxir	CPT1a	Fatty acid oxidation	(131)	*In vitro*	Increased sensitivity to ATO & apoptosis
LCL204	Acid ceramidase	Sphingolipids	(132)	*In vivo*	Increased overall survival of PDXs & decreased leukaemic burden
Statins: lovastatin, pravastatin	HMG-CoA reductase	Mevalonate biosynthesis	(133)	Phase I/II	Increased sensitivity to Venetoclax
ST1326	CPT1a	Fatty acid oxidation	(134)	*In vitro* & *in vivo*	Growth arrest & induced apoptosis

6-AN, 6-aminonicotinamide; 2-DG, 2-deoxy-D-glucose; 2-D-HG, 2-D-hydroxyglutarate; Bap, a combination of lipid-regulating bezafibrate (BEZ) and the sex hormone medroxyprogesterone acetate; CKMT1, creatine kinase mitochondrial 1; CPT1A, carnitine palmitoyltransferase 1A; DHODH, dihydroorotate dehydrogenase; ETC, electron transport chain; IDH1, isocitrate dehydrogenase 1; mTOR, mechanistic target of rapamycin kinase; HMG, hydroxy methylglutaryl; OXPHOS, oxidative phosphorylation; PPP, pentose phosphate pathway.

### Targeting Glucose Metabolism

As outlined above, the reliance of AML blasts on glucose metabolism makes it an attractive target for AML therapy. Unsurprisingly, inhibition of glycolytic enzymes has been shown to suppress glycolysis, leading to significant energy loss and leukaemia cell death (38). Overexpression of GLUT1 is associated with poor chemotherapy response in patients with AML and inhibition of GLUT1 is considered to be a promising treatment strategy (43). Inhibition of the glycolysis rate-limiting enzyme hexokinase-2 with 2-Deoxy-D-glucose (2-DG) significantly improved the cytotoxicity of Ara-C in primary AML blasts and cell lines (37). 2-DG is a synthetic glucose analogue that not only perturbs glucose metabolism, but also interferes with OXPHOS, depleting cellular energy, N-linked glycosylation, and autophagy induction (135). AML primary blasts and cell lines harbouring c-KIT and FLT3-ITD mutations showed sensitivity to 2-DG treatment, compared with normal haematopoietic cells (112).

Direct inhibition of hexokinase-2 with 3-bromopyruvate is also considered as another treatment strategy in AML cells with high glycolytic activity, such as HL-60 cells (43). Also, AML cells with FLT3-ITD showed resistance to sorafenib treatment, a kinase inhibitor for the treatment of FLT-ITD+ cells, but were sensitive to hexokinase-2 inhibitors including 2-DG and 3-bromopyruvic acid (113). Inhibiting glycolysis is mainly investigated in AML patients at diagnosis or in the bulk of the AML population and further investigations in relapsed AML and LSCs are needed.

### Targeting Mitochondria-Related Pathways

Inhibition of different mitochondrial metabolic processes such as OXPHOS and aerobic respiration are emerging treatment strategies in patients with AML. An ongoing phase I clinical trial is using a selective and potent OXPHOS inhibitor, IACS-010759, in treating patients with relapsed/refractory AML (ClinicalTrials.gov Identifier NCT02882321). The anti-leukaemic activity of IACS-010759 is mediated by the AMPK pathway in OXPHOS-dependent primary AML blasts and cell lines (136). In sensitive primary AML blasts with high levels of AMPK, IACS-010759 induced AMPK activation leading to mTOR suppression and reduced cell growth. In contrast, resistant primary AML blasts with low AMPK expression were less responsive to the treatment (136). mTORC1 suppression enforces primary AML blasts to reprogram from glycolysis to OXPHOS by promoting the TCA cycle and this has led to the use of mTORC1 inhibitor (rapamycin) and OXPHOS inhibitor (metformin) in targeting resistant blast cells (38). Another drug that targets the ETC is Brequinar sodium which suppresses the inner mitochondrial membrane protein dihydroorotate dehydrogenase, playing an important role in pyrimidine biosynthesis (137). Inhibition of dihydroorotate dehydrogenase with Brequinar induced myeloid differentiation and apoptosis of primary AML blasts (114, 115).

The pyruvate dehydrogenase complex is responsible for catalysing the carboxylation of pyruvate to acetyl-CoA. The pyruvate dehydrogenase complex acts as a central hub between three different metabolic pathways including glycolysis, the TCA cycle, and OXPHOS (138). Hampering the pyruvate dehydrogenase complex with a selective inhibitor dichloroacetate has led to increased OXPHOS, ROS content, and activated antioxidant response which activates DNA repair genes (138). Therefore, applying dichloroacetate in combination with genotoxic drugs including doxorubicin and Ara-C induced ROS generation, DNA damage and apoptosis in AML cells (139, 140). A study by Emadi et al. found that sequential administration of dichloroacetate in combination with arsenic trioxide exerted synergistic anti-leukaemic effects in primary blasts from AML patients and AML cell lines with FLT3-ITD (139).

Another mitochondrial-related treatment approach is the exposure of AML cells to BCL-2 inhibitors. Venetoclax is a potent BCL-2 inhibitor that has shown activity in haematologic malignancies including both as a single agent or in combination with chemotherapeutics agents (116, 141). Treatment of 32 relapsed/refractory AML patients with venetoclax led to a favourable response in 19% of patients while another 19% of patients showed a modest anti-leukaemic response (116). Likewise, in a small-scale study of seven patients with secondary AML, two patients achieved complete remission with venetoclax monotherapy (142). However, combination therapy of venetoclax has been demonstrated to be much more efficient than single-agent treatment (117, 118). The combination treatment of elderly patients with venetoclax and azacitidine led to 36.7% of patients achieving complete remission compared to 17.9% of patients in the control group (118). In a similar study, venetoclax combined with the FLT3 inhibitor quizartinib showed synergistic anti-leukaemic activity in cell lines and primary patient samples, and prolonged the survival of FLT-ITD+ AML mice, compared to the modest effects of single-agent treatments (119).

Venetoclax in combination with statins has also been proven to induce synergistic anti-leukaemic effects. It was reported that statins increased venetoclax efficacy in AML which is mediated by protein geranyl-geranylation, BCL-2 modulation, and upregulation of pro-apoptotic genes such as PUMA (133). Moreover, the FAO inhibitor etomoxir was shown to increase the therapeutic efficacy of another BCL-2 inhibitor ABT-737 *in vivo* (143). On the other hand, a recent genome-wide CRISPR/Cas9 screen and metabolomics study revealed heme biosynthesis as a key regulator of drug sensitivity. AML cells treated with venetoclax have upregulated heme biosynthesis which is an important regulator of mitochondrial-related apoptosis through ETC and OXPHOS (62). In line with these studies, Jones et al. indicated that both depleting amino acid in the culture media and inhibition of BCL-2 with venetoclax significantly reduced OXPHOS and selectively reduced the viability of ROS-low LSCs *ex vivo* and sensitised them to azacitidine treatment (18).

As discussed above, AML cells with IDH1/2 mutations have a higher amount of 2-D-hydroxyglutarate, which blocks differentiation. Targeting AML cells with mutant IDH1/2 profiles has resulted in promising anti-leukaemic effects in patients and cell lines. Single-agent treatment of relapsed/refractory AML patients with enasidenib, a selective IDH2 inhibitor, had an overall response rate of 40.3% (122). Co-occurring mutations in NRAS and MAPK pathways were observed in nonresponding patients which were consistent with the role of RAS signalling in primary therapeutic resistance (123). Moreover, treatment of mutant IDH2 cells with enasidenib suppressed 2-D-hydroxyglutarate production and induced cellular differentiation in primary AML cells and xenograft models (124). Enasidenib also increased survival rates in IDH2-mutant AML xenografts which supported the initiation of clinical trials of enasidenib in patients with haematologic malignancies with IDH2 mutations (ClinicalTrials.gov Identifier: NCT01915498).

Aside from suppression of mitochondria-related pathways, direct perturbation of mitochondrial components has also been shown to be another attractive therapeutic strategy and its significance is increasingly being recognised in hematologic malignancies including AML. For example, blockade of translation with tigecycline has led to a significant antileukaemic effect against primary AML cells. Tigecycline is a glycylcycline class antibiotic that binds to the 30S ribosomal subunit and perturbs mitochondrial translation. It selectively destroyed primary bulk AML cells and CD34^+^CD38^-^ LSCs without affecting normal haematopoietic cells (73). Inhibition of a mitochondrial caseinolytic protein protease with A2-32-01 has led to promising results in AML (74). A2-32-01 is an anti-virulence drug that specifically inhibited ClpP and eliminated both AML cell lines and primary samples with elevated ClpP expression without killing normal haematopoietic cells (74). Suppression of mtDNA replication with 2’,3’-dideoxy-cytidine is also effective in the treatment of AML patients with high mtDNA polymerase activity. 2’,3’-dideoxy-cytidine is a nucleoside analogue that selectively inhibits mtDNA polymerase, perturbs OXPHOS and induces cytotoxicity in AML cell lines and primary samples while sparing normal haematopoietic cells (121). 2’,3’-dideoxy-cytidine also decreases mtDNA, ETC proteins and induces tumour regression without toxicity *in vivo* and selectively targeted LSCs with leukaemia initiating potential in secondary xenografts (121). Overall, these observations emphasise the potential of targeting mitochondrial related pathways to improve AML treatment outcomes.

### Targeting Amino Acid Metabolism

Significant glutamine uptake is a common metabolic feature of AML cells (60, 62) and unsurprisingly inhibition of glutamine uptake or function is a potent strategy to treat AML. GLS1 is an important enzyme to increase glutamine levels and its overexpression is reported to be involved in the drug resistance of AML cells. Depletion of glutamine in the culture media or inhibition of glutamine biosynthesis by GLS1 knockdown has been shown to suppress the growth of primary AML cells and cell lines (144). Moreover, inhibition of glutamine metabolism by a GLS1 inhibitor (CB-839) perturbs GSH production in AML cell lines, leading to the elevation of ROS and apoptotic cell death (145). Moreover, GLS1 inhibition made AML cells susceptible to chemotherapeutic drugs such as arsenic trioxide (ATO) that synergistically perturbed mitochondrial redox state and induced apoptosis in AML cell lines, primary samples and *in vivo* models (145). Suppression of GLS1 with CB-839 activated mitochondrial apoptosis and depleted glutamine in the culture of AML cell lines which synergises with the anti-leukaemic effects of venetoclax (78). A CRISPR/Cas9 screen of MOLM-13 AML cells treated with FLT3 inhibitors revealed that GLS1 mediates resistance to tyrosine kinase inhibitors (76). Concomitant exposure of FLT3-ITD AML cell lines to quizartinib (FLT3 inhibitor) and CB-839 potently decreased viability and enhanced the overall survival of immune-deficient mice engrafted with AML patient-derived xenografts implying the dependence of AML cells on glutamine metabolism for drug resistance (146).

Recent studies have highlighted the association of amino acid metabolism with OXPHOS in AML cells. Pharmacological inhibition of amino acid metabolism with venetoclax reduced OXPHOS and induced cell death in *de novo* ROS-low LSCs (18). In the same study, it was shown that ROS-low LSCs derived from patients with relapsed AML were able to resist venetoclax as they are not reliant on amino acid metabolism and supply their energy through fatty acid metabolism (18). Moreover, comprehensive metabolomics analysis of relapsed ROS-low LSCs indicated high levels of nicotinamide and NAD+, which play an important role in sustaining OXPHOS. Therefore, perturbation of nicotinamide metabolism selectively targeted and eliminated relapsed ROS-low LSCs, highlighting the value of targeting amino acid metabolism (147). Another study has indicated that ROS-low LSCs metabolise exogenous cysteine to GSH which, in turn, activates succinate dehydrogenase a key component of mitochondrial ETC complex II. The same study showed that cysteine depletion impaired GSH synthesis disrupted ETC, which eventually inhibited OXPHOS and ATP production leading to ROS-low LSC death (148).

Asparagine is another important amino acid in AML metabolism which is generated by glutamine and aspartate (149). L-asparaginase converts asparagine to aspartic acid and has shown potent anti-leukaemic activity and has been used for the treatment of patients with *de novo* and relapsed AML (125, 126). The combination of L-asparaginase with high-dose Ara-C and mitoxantrone has resulted in positive outcomes in AML patients with an aberrant asparagine metabolism (125, 150). These results have led to the initiation of a randomised phase 2b trial which evaluates the efficacy of ERY001, L-asparaginase encapsulated in red blood cells, in elderly AML patients who are unfit for intensive chemotherapy (127).

Lastly, inhibition of arginine has resulted in promising results in AML treatment. A study by Miraki-Moud et al. demonstrated that AML blasts obtained from patients at diagnosis relied on arginine to survive and proliferate (81). Decreased arginosuccinate synthetase-I activity with a pegylated arginine deiminase called ADI-PEG 20 showed anti-leukaemic activity *in vitro* and *in vivo* with minor toxicity on normal haematopoietic cells (81). Depletion of arginine with another pegylated recombinant arginase, BCT-100, suppressed AML blast cell proliferation and reduced leukaemia engraftment *in vivo* (151). In addition, single-agent BCT-100 caused significant cell death in adult AML blasts and synergised with cytarabine activity (151). Likewise, an ongoing study is further testing the safety and efficacy of BCT-100 in younger patients with relapsed/refractory leukaemia (ClinicalTrials.gov Identifier NCT03455140). Together these studies demonstrate that the targeting of amino acid metabolism, much like glycolysis and mitochondrial metabolism, is a promising strategy for the treatment of AML.

### Targeting Lipid Metabolism

Increased FA synthesis and FAO are two deregulated metabolic pathways that support leukemogenesis. Unsurprisingly FA synthesis and FAO are therefore attractive targets for inhibition (84, 152). Inhibition of FA synthesis with CPI-613 synergised with the anti-leukaemic effects of doxorubicin in cell line-derived AML xenografts and extended the median survival from 12 days with doxorubicin alone to 16 days with the combination of both (130).

Likewise, FAO suppression has been shown to inhibit AML. Suppressing FAO by a specific CPT1A inhibitor, ST1326, reduced cell growth, induced apoptosis, and had favourable interaction with the cytotoxic effects of Ara-C in AML cell lines (134). In a similar study, inhibition of CPT1A with etomoxir synergised with arsenic trioxide and caused anti-leukaemic activity in AML cell lines. Etomoxir inhibits carnitine palmitoyl transferase 1 activity thus, hindering fatty acid transport into mitochondria and β-oxidation (131). Moreover, blockade of CPT1A with avocatin B selectively targeted AML blasts while preserving normal HSCs through decreased NADPH levels and increased DNA damage-related cell death (129). More interestingly, concomitant treatment of LSCs from patients at relapse with azacitidine and venetoclax resulted in only a minor reduction of viability due to increased FAO as a resistance mechanism (18). Suppression of FA uptake by a CD36 inhibitor, sorbitan sesquioleate, re-sensitised ROS-low LSCs from patients at relapse to azacitidine (18). Another study has suggested that LSCs residing in the BMME are enriched in adipose tissue and are protected by lipolysis or FAO. CD36^+^CD34^+^ LSCs have a higher FAO rate compared to differentiated leukaemia cells or normal HSCs. Therefore, targeting FAO might selectively target and eliminate the CD36^+^CD34^+^ LSC population (87).

Targeting sphingolipids has recently been suggested as a novel avenue for AML therapy (99, 100). Acid ceramidase catalyses a reaction that generates sphingolipids, contributing to AML blast survival *via* upregulation of the anti-apoptotic protein MCL-1. The acid ceramidase inhibitor LCL204 reduced the leukaemic burden in NSG mice engrafted with primary AML cells (132). Consistent with this finding, inhibition of ceramide in AML cells harbouring FLT3-ITD sensitised them to FLT3 inhibitors, which provides an opportunity to target resistant primary AML cells with FLT3 mutations (153). Treatment of AML cell lines, primary blasts and CD34^+^CD38^−^CD123^+^ LSCs with MP-A08, a selective sphingosine kinase inhibitor, significantly induced apoptosis, with negligible effects on normal HSCs from healthy samples (99). Furthermore, exposure of AML patient-derived xenografts to MP-A08 reduced tumour burden and prolonged mouse survival without affecting normal murine haematopoiesis (99).

Compared to glycolytic, mitochondrial, and amino acid metabolism, the role of FAO and FA biosynthesis is more important in normal HSCs and LSCs than in the bulk of leukaemic cells. These findings suggest that AML progenitor cells utilise lipid metabolism to survive and that targeting these pathways is a viable future therapeutic strategy for the elimination of these cells.

## Conclusion

In the past decade, extensive efforts have been made to uncover the main metabolic features of tumour cells compared to their normal counterparts (6, 9). These investigations have suggested that some cancers, such as leukaemia, are driven by metabolic alterations and thus appropriate metabolic-based treatment strategies are needed (32, 33, 154). Moreover, the unique metabolic signature of leukaemic cells could be used for monitoring disease progression and prognosis.

The majority of previous research on AML metabolism has suggested that leukaemic blasts can compensate for energy shortage and adapt to new metabolic programs during disease progression and chemotherapy. However, perturbing metabolic pathways in leukaemic blasts alone is not efficient enough to improve clinical outcomes and reduce the relapse rate in patients. This stems from the fact that previous studies mainly focused on leukaemic blasts and not LSCs, and the latter are the main mediators of AML relapse (3).

A major limitation is the lack of clinically relevant *in vivo* models to study AML metabolism (155). Clinically relevant mouse models are required to improve the efficacy and correlation between *in vitro* and *in vivo* experiments (155). For example, discrepancy of *in vitro* and *in vivo* models is evident in the growth of AML cells in human plasma-like medium (HPLM). HPLM contains biologically relevant levels of uric acid which inhibits *de novo* pyrimidine synthesis. Uric acid levels are higher in human blood than in mice serum and therefore can inhibit uridine monophosphate synthase, a crucial enzyme for pyrimidine synthesis. This has been shown to reduce the sensitivity of AML cells to chemotherapeutic drugs such as 5-fluorouracil and highlights the importance of the tumour microenvironment on cell metabolism and the efficacy of drugs (156).

Another limiting issue is the lack of efficient culture media to accurately represent the tumour environment in patients (155). Extensive research has been done to supplement culture media with nutrients that are crucial in human serum and this has led to the use of enriched mediums such as HPLM and Plasmax to better represent the biological conditions of patients (156, 157). Furthermore, cell co-cultures and 3D cultures provide a tumour microenvironment which is an important regulator of drug response and metabolic reprogramming *in vitro* and therefore these cultures better represent the cellular environment of patients. As we mentioned in this review, adipose tissues and adipocytes can modulate drug response in AML and provide compensatory nutrients for leukaemic cells to resist chemotherapy. Therefore, it is unsurprising that co-culturing AML cells with bone marrow adipocytes noticeably induced resistance against CPT1a inhibitors and increased FAO (143, 158). Treatment of AML cells with CPT1a inhibitor significantly increased free FAs and glucose uptake specifically in the co-cultures with bone marrow adipocytes; underlining the vital role of the microenvironment in regulating energy demands and chemotherapy response (158). Therefore, the lack of plasma and blood metabolites in cell culture could impair the efficacy of metabolic inhibitors and question the clinical translatability of many *in vitro* approaches.

Patient and cellular heterogeneity is another level of complexity in investigating AML metabolism and identifying effective treatment strategies. Previous research has shown that AML cells evade chemotherapy by altering their gene expression pattern which leads to rewired metabolism (22, 120). In fact, AML patients treated with Ara-C and metabolic inhibitors showed altered metabolic and transcriptional regulation. It has also been shown that the antiproliferative effect of metabolic inhibitors differs across patients and the AML cells derived from different patients are metabolically heterogenic, likely due to genomic heterogeneity (22). In another study, the comparison of the gene expression profile of chemotherapy responders and non-responders showed a significant level of genome heterogeneity which was linked to altered cellular pathways including metabolism (120). This level of genome and metabolome heterogeneity reinforces the notion that a “one-size-fits-all” strategy would not be effective for AML therapy and therefore, personalised medicine is required to tailor treatments to achieve the best outcome for individual patients (159). Nevertheless, targeting metabolic pathways in AML is a promising therapeutic approach and a rapidly emerging field that requires significant attention. Therefore, further *in vivo* studies distinguishing metabolic requirements of normal HSCs, AML blasts, and LSCs are necessary to design efficient treatment regimens for patients with AML at different stages of the disease including diagnosis, relapse and remission.

In this review, we provide a timely update on the latest findings of dysregulated metabolic pathways in HSCs, LSCs and AML blasts and how these pathways can be targeted for better treatment outcomes. Previous literature has shown that AML is mainly driven by LSCs and targeting these cells would be an effective treatment strategy for AML. Residing in the BMME allows LSCs to adapt their metabolic profile, evade chemotherapy and drive disease relapse. Conventional chemotherapy fails to selectively eliminate LSCs, is almost ineffective to perturb metabolic pathways, and is toxic to normal HSCs. Therefore, targeting metabolic pathways can re-sensitise AML blasts and LSCs to conventional chemotherapy while sparing healthy haematopoietic cells and normal HSCs. Finally, AML is genetically, and metabolically heterogeneous and *in vitro* experiments do not reflect what might occur *in vivo* and in patients. Therefore, further studies are required to effectively increase survival and enhance treatment outcomes in patients.

## Author Contributions

YM, RL and PC were primarily responsible for the manuscript. All authors contributed to editing and reviewing content for the manuscript and approved the final version.

## Funding

This work was supported by Tour de Cure Pioneering Grant (Tour De Cure, Australia) [Grant #: RSP-00122-19/20] to PC and National Health and Medical Research Council of Australia [NHMRC Fellowships APP1059804 and APP1157871] to RBL. We acknowledge the Research Training Program Scholarship (RTP) awarded to YM for study towards a PhD in the Faculty of Medicine. Figures were created with BioRender.com.

## Conflict of Interest

The authors declare that the research was conducted in the absence of any commercial or financial relationships that could be construed as a potential conflict of interest.

## Publisher’s Note

All claims expressed in this article are solely those of the authors and do not necessarily represent those of their affiliated organizations, or those of the publisher, the editors and the reviewers. Any product that may be evaluated in this article, or claim that may be made by its manufacturer, is not guaranteed or endorsed by the publisher.

## Glossary

**Table:**

6-AN	6-aminonicotinamide
2-DG	2-Deoxy-D-glucose
2-D-HG	2-D-hydroxyglutarate
α-KG	α-ketoglutarate
AAs	Amino acids
ABT-199	Venetoclax
ABC	ATP-Binding Cassette
Akt	Protein kinase B
ALAT	Alanine transferase
AML	Acute myeloid leukaemia
AMPK	AMP-activated protein kinase
Bap	A combination of lipid-regulating bezafibrate (BEZ) and the sex hormone medroxyprogesterone acetate
BCAT1	branched-chain amino acid transaminase 1
BMME	Bone marrow microenvironment
Cas9	CRISPR-associated protein 9
CD	Cluster of differentiation
c-KIT	Cluster of differentiation 117
CKMT1	Creatine kinase mitochondrial 1
ClpP	ATP-dependent Clp protease
CoA	Coenzyme A
CPT1A	Carnitine palmitoyltransferase 1A
CRISPR	Clustered regularly interspaced short palindromic repeats
Cytarabine	Ara-C
DHODH	Dihydroorotate dehydrogenase
ETC	Electron transport chain
F1P	Fructose-1-phosphate
FADH2	Flavin adenine dinucleotide
FLT3-ITD	Fms-like tyrosine kinase-3 internal tandem duplication
G3P	Glycerol-3-phosphate
G6P	Glucose-6-phosphate
GLS	Glutaminase
GLUTs	Glucose transporters
GPX1	Glutathione peroxidase 1
GSH	Glutathione
GSR	Glutathione-disulfide reductase
HIF-1α	Hypoxia-inducible factor 1-alpha
HMG	Hydroxy methylglutaryl
HPLM	Human plasma-like medium
HSCs	Haematopoietic stem cells
IDH	Isocitrate dehydrogenase
LDHA	Lactate dehydrogenase
LSCs	Leukaemic stem cells
MCL-1	Myeloid Cell Leukaemia 1
mtDNA	Mitochondrial DNA
mTOR	Mechanistic target of rapamycin
NAD	Nicotinamide adenine dinucleotide
NADPH	Nicotinamide adenine dinucleotide phosphate
NSG	NOD scid gamma mouse
OAA	Oxaloacetate
OXPHOS	Oxidative phosphorylation
PEP	Phosphoenolpyruvate
PI3K	Phosphoinositide 3-kinase
PKM2	Pyruvate kinase M2
PPP	Pentose phosphate pathway
PUMA	p53 upregulated modulator of apoptosis
R5P	Ribose-5-phosphate
ROS	Reactive oxygen species
shRNA	Short or small hairpin RNA
SLCs	Solute carriers
TCA	Tricarboxylic acid

## References

[1] Dong Y Shi O Zeng Q Lu X Wang W Li Y. Leukemia Incidence Trends at the Global, Regional, and National Level Between 1990 and 2017. Exp Hematol Oncol (2020) 9(1):14. doi: 10.1186/s40164-020-00170-6 32577323 PMC7304189

[2] Short NJ Rytting ME Cortes JE. Acute Myeloid Leukaemia. Lancet (2018) 392(10147):593–606. doi: 10.1016/S0140-6736(18)31041-9 30078459 PMC10230947

[3] Thomas D Majeti R. Biology and Relevance of Human Acute Myeloid Leukemia Stem Cells. Blood (2017) 129(12):1577–85. doi: 10.1182/blood-2016-10-696054 PMC5364335 28159741

[4] Dombret H Gardin C. An Update of Current Treatments for Adult Acute Myeloid Leukemia. Blood (2016) 127(1):53–61. doi: 10.1182/blood-2015-08-604520 26660429 PMC4705610

[5] Rashidi A Walter RB Tallman MS Appelbaum FR DiPersio JF. Maintenance Therapy in Acute Myeloid Leukemia: An Evidence-Based Review of Randomized Trials. Blood (2016) 128(6):763–73. doi: 10.1182/blood-2016-03-674127 PMC4982451 27354720

[6] Lehuede C Dupuy F Rabinovitch R Jones RG Siegel PM. Metabolic Plasticity as a Determinant of Tumor Growth and Metastasis. Cancer Res (2016) 76(18):5201–8. doi: 10.1158/0008-5472.CAN-16-0266 27587539

[7] Du W Amarachintha S Wilson AF Pang Q. SCO2 Mediates Oxidative Stress-Induced Glycolysis to Oxidative Phosphorylation Switch in Hematopoietic Stem Cells. Stem Cells (2016) 34(4):960–71. doi: 10.1002/stem.2260 PMC4838537 26676373

[8] Ito K Bonora M Ito K. Metabolism as Master of Hematopoietic Stem Cell Fate. Int J Hematol (2019) 109(1):18–27. doi: 10.1007/s12185-018-2534-z 30219988 PMC6318064

[9] Kreitz J Schonfeld C Seibert M Stolp V Alshamleh I Oellerich T. Metabolic Plasticity of Acute Myeloid Leukemia. Cells (2019) 8(8):805. doi: 10.3390/cells8080805 PMC6721808 31370337

[10] Velten L Haas SF Raffel S Blaszkiewicz S Islam S Hennig BP. Human Haematopoietic Stem Cell Lineage Commitment is a Continuous Process. Nat Cell Biol (2017) 19(4):271–81. doi: 10.1038/ncb3493 PMC5496982 28319093

[11] Lapidot T Sirard C Vormoor J Murdoch B Hoang T Caceres-Cortes J. A Cell Initiating Human Acute Myeloid Leukaemia After Transplantation Into SCID Mice. Nature (1994) 367(6464):645–8. doi: 10.1038/367645a0 7509044

[12] Ye H Adane B Khan N Sullivan T Minhajuddin M Gasparetto M. Leukemic Stem Cells Evade Chemotherapy by Metabolic Adaptation to an Adipose Tissue Niche. Cell Stem Cell (2016) 19(1):23–37. doi: 10.1016/j.stem.2016.06.001 27374788 PMC4938766

[13] Velten L Story BA Hernandez-Malmierca P Raffel S Leonce DR Milbank J. Identification of Leukemic and Pre-Leukemic Stem Cells by Clonal Tracking From Single-Cell Transcriptomics. Nat Commun (2021) 12(1):1366. doi: 10.1038/s41467-021-21650-1 33649320 PMC7921413

[14] Herrmann H Sadovnik I Eisenwort G Rulicke T Blatt K Herndlhofer S. Delineation of Target Expression Profiles in CD34+/CD38- and CD34+/CD38+ Stem and Progenitor Cells in AML and CML. Blood Adv (2020) 4(20):5118–32. doi: 10.1182/bloodadvances.2020001742 PMC7594398 33085758

[15] Hope KJ Jin L Dick JE. Acute Myeloid Leukemia Originates From a Hierarchy of Leukemic Stem Cell Classes That Differ in Self-Renewal Capacity. Nat Immunol (2004) 5(7):738–43. doi: 10.1038/ni1080 15170211

[16] Chopra M Bohlander SK. The Cell of Origin and the Leukemia Stem Cell in Acute Myeloid Leukemia. Genes Chromosomes Cancer (2019) 58(12):850–8. doi: 10.1002/gcc.22805 31471945

[17] Zhou J Chng WJ. Identification and Targeting Leukemia Stem Cells: The Path to the Cure for Acute Myeloid Leukemia. World J Stem Cells (2014) 6(4):473–84. doi: 10.4252/wjsc.v6.i4.473 PMC4172676 25258669

[18] Jones CL Stevens BM D'Alessandro A Reisz JA Culp-Hill R Nemkov T. Inhibition of Amino Acid Metabolism Selectively Targets Human Leukemia Stem Cells. Cancer Cell (2018) 34(5):724–40.e4. doi: 10.1016/j.ccell.2018.10.005 30423294 PMC6280965

[19] Lagadinou ED Sach A Callahan K Rossi RM Neering SJ Minhajuddin M. BCL-2 Inhibition Targets Oxidative Phosphorylation and Selectively Eradicates Quiescent Human Leukemia Stem Cells. Cell Stem Cell (2013) 12(3):329–41. doi: 10.1016/j.stem.2012.12.013 PMC3595363 23333149

[20] Agarwal A Bolosky WJ Wilson DB Eide CA Olson SB Fan G. Differentiation of Leukemic Blasts is Not Completely Blocked in Acute Myeloid Leukemia. Proc Natl Acad Sci USA (2019) 116(49):24593–9. doi: 10.1073/pnas.1904091116 PMC6900505 31754026

[21] van Galen P Hovestadt V Wadsworth Ii MH Hughes TK Griffin GK Battaglia S. Single-Cell RNA-Seq Reveals AML Hierarchies Relevant to Disease Progression and Immunity. Cell (2019) 176(6):1265–81.e24. doi: 10.1016/j.cell.2019.01.031 30827681 PMC6515904

[22] Gronningsaeter IS Reikvam H Aasebo E Bartaula-Brevik S Tvedt TH Bruserud O. Targeting Cellular Metabolism in Acute Myeloid Leukemia and The Role of Patient Heterogeneity. Cells (2020) 9(5):1155. doi: 10.3390/cells9051155 PMC7290417 32392896

[23] Zhou HS Carter BZ Andreeff M. Bone Marrow Niche-Mediated Survival of Leukemia Stem Cells in Acute Myeloid Leukemia: Yin and Yang. Cancer Biol Med (2016) 13(2):248–59. doi: 10.20892/j.issn.2095-3941.2016.0023 PMC4944541 27458532

[24] Wang A Zhong H. Roles of the Bone Marrow Niche in Hematopoiesis, Leukemogenesis, and Chemotherapy Resistance in Acute Myeloid Leukemia. Hematology (2018) 23(10):729–39. doi: 10.1080/10245332.2018.1486064 29902132

[25] Haltalli MLR Lo Celso C. Targeting Adhesion to the Vascular Niche to Improve Therapy for Acute Myeloid Leukemia. Nat Commun (2020) 11(1):3691. doi: 10.1038/s41467-020-17594-7 32703951 PMC7378234

[26] Tabe Y Konopleva M. Role of Microenvironment in Resistance to Therapy in AML. Curr Hematol Malig Rep (2015) 10(2):96–103. doi: 10.1007/s11899-015-0253-6 25921386 PMC4447522

[27] Ito K Suda T. Metabolic Requirements for the Maintenance of Self-Renewing Stem Cells. Nat Rev Mol Cell Biol (2014) 15(4):243–56. doi: 10.1038/nrm3772 PMC4095859 24651542

[28] Jang YY Sharkis SJ. A Low Level of Reactive Oxygen Species Selects for Primitive Hematopoietic Stem Cells That may Reside in the Low-Oxygenic Niche. Blood (2007) 110(8):3056–63. doi: 10.1182/blood-2007-05-087759 PMC2018677 17595331

[29] Ishikawa F Yoshida S Saito Y Hijikata A Kitamura H Tanaka S. Chemotherapy-Resistant Human AML Stem Cells Home to and Engraft Within the Bone-Marrow Endosteal Region. Nat Biotechnol (2007) 25(11):1315–21. doi: 10.1038/nbt1350 17952057

[30] Forte D Garcia-Fernandez M Sanchez-Aguilera A Stavropoulou V Fielding C Martin-Perez D. Bone Marrow Mesenchymal Stem Cells Support Acute Myeloid Leukemia Bioenergetics and Enhance Antioxidant Defense and Escape From Chemotherapy. Cell Metab (2020) 32(5):829–43 e9. doi: 10.1016/j.cmet.2020.09.001 32966766 PMC7658808

[31] Hole PS Zabkiewicz J Munje C Newton Z Pearn L White P. Overproduction of NOX-Derived ROS in AML Promotes Proliferation and is Associated With Defective Oxidative Stress Signaling. Blood (2013) 122(19):3322–30. doi: 10.1182/blood-2013-04-491944 24089327

[32] Chapuis N Poulain L Birsen R Tamburini J Bouscary D. Rationale for Targeting Deregulated Metabolic Pathways as a Therapeutic Strategy in Acute Myeloid Leukemia. Front Oncol (2019) 9:405. doi: 10.3389/fonc.2019.00405 31192118 PMC6540604

[33] Stuani L Sabatier M Sarry JE. Exploiting Metabolic Vulnerabilities for Personalized Therapy in Acute Myeloid Leukemia. BMC Biol (2019) 17(1):57. doi: 10.1186/s12915-019-0670-4 31319822 PMC6637566

[34] Castro I Sampaio-Marques B Ludovico P. Targeting Metabolic Reprogramming in Acute Myeloid Leukemia. Cells (2019) 8(9):976. doi: 10.3390/cells8090967 PMC6770240 31450562

[35] Mistry JJ Hellmich C Moore JA Marlein CR Pillinger G Collins A. Daratumumab Inhibits AML Metabolic Capacity and Tumor Growth Through Inhibition of CD38 Mediated Mitochondrial Transfer From Bone Marrow Stromal Cells to Blasts in the Leukemic Microenvironment. Blood (2019) 134(Supplement_1):1385. doi: 10.1182/blood-2019-128592

[36] Simsek T Kocabas F Zheng J Deberardinis RJ Mahmoud AI Olson EN. The Distinct Metabolic Profile of Hematopoietic Stem Cells Reflects Their Location in a Hypoxic Niche. Cell Stem Cell (2010) 7(3):380–90. doi: 10.1016/j.stem.2010.07.011 PMC4159713 20804973

[37] Chen WL Wang JH Zhao AH Xu X Wang YH Chen TL. A Distinct Glucose Metabolism Signature of Acute Myeloid Leukemia With Prognostic Value. Blood (2014) 124(10):1645–54. doi: 10.1182/blood-2014-02-554204 PMC5726328 25006128

[38] Poulain L Sujobert P Zylbersztejn F Barreau S Stuani L Lambert M. High Mtorc1 Activity Drives Glycolysis Addiction and Sensitivity to G6PD Inhibition in Acute Myeloid Leukemia Cells. Leukemia (2017) 31(11):2326–35. doi: 10.1038/leu.2017.81 28280275

[39] Ye H Adane B Khan N Alexeev E Nusbacher N Minhajuddin M. Subversion of Systemic Glucose Metabolism as a Mechanism to Support the Growth of Leukemia Cells. Cancer Cell (2018) 34(4):659–73 e6. doi: 10.1016/j.ccell.2018.08.016 30270124 PMC6177322

[40] Panuzzo C Jovanovski A Pergolizzi B Pironi L Stanga S Fava C. Mitochondria: A Galaxy in the Hematopoietic and Leukemic Stem Cell Universe. Int J Mol Sci (2020) 21(11):3928. doi: 10.3390/ijms21113928 PMC7312164 32486249

[41] Liberti MV Locasale JW. The Warburg Effect: How Does it Benefit Cancer Cells? Trends Biochem Sci (2016) 41(3):211–8. doi: 10.1016/j.tibs.2015.12.001 PMC4783224 26778478

[42] Hay N. Reprogramming Glucose Metabolism in Cancer: Can it be Exploited for Cancer Therapy? Nat Rev Cancer (2016) 16(10):635–49. doi: 10.1038/nrc.2016.77 PMC5516800 27634447

[43] Song K Li M Xu X Xuan LI Huang G Liu Q. Resistance to Chemotherapy is Associated With Altered Glucose Metabolism in Acute Myeloid Leukemia. Oncol Lett (2016) 12(1):334–42. doi: 10.3892/ol.2016.4600 PMC4906727 27347147

[44] Wang YH Israelsen WJ Lee D Yu VWC Jeanson NT Clish CB. Cell-State-Specific Metabolic Dependency in Hematopoiesis and Leukemogenesis. Cell (2014) 158(6):1309–23. doi: 10.1016/j.cell.2014.07.048 PMC4197056 25215489

[45] Mirabilii S Ricciardi MR Piedimonte M Gianfelici V Bianchi MP Tafuri A. Biological Aspects of mTOR in Leukemia. Int J Mol Sci (2018) 19(8):2396. doi: 10.3390/ijms19082396 PMC6121663 30110936

[46] Jones CL Inguva A Jordan CT. Targeting Energy Metabolism in Cancer Stem Cells: Progress and Challenges in Leukemia and Solid Tumors. Cell Stem Cell (2021) 28(3):378–93. doi: 10.1016/j.stem.2021.02.013 PMC7951949 33667359

[47] Saito Y Chapple RH Lin A Kitano A Nakada D. AMPK Protects Leukemia-Initiating Cells in Myeloid Leukemias From Metabolic Stress in the Bone Marrow. Cell Stem Cell (2015) 17(5):585–96. doi: 10.1016/j.stem.2015.08.019 PMC4597792 26440282

[48] Bertacchini J Guida M Accordi B Mediani L Martelli AM Barozzi P. Feedbacks and Adaptive Capabilities of the PI3K/Akt/mTOR Axis in Acute Myeloid Leukemia Revealed by Pathway Selective Inhibition and Phosphoproteome Analysis. Leukemia (2014) 28(11):2197–205. doi: 10.1038/leu.2014.123 24699302

[49] Kharas MG Okabe R Ganis JJ Gozo M Khandan T Paktinat M. Constitutively Active AKT Depletes Hematopoietic Stem Cells and Induces Leukemia in Mice. Blood (2010) 115(7):1406–15. doi: 10.1182/blood-2009-06-229443 PMC2826762 20008787

[50] Allegretti M Ricciardi MR Licchetta R Mirabilii S Orecchioni S Reggiani F. The Pan-Class I Phosphatidyl-Inositol-3 Kinase Inhibitor NVP-BKM120 Demonstrates Anti-Leukemic Activity in Acute Myeloid Leukemia. Sci Rep (2015) 5:18137. doi: 10.1038/srep18137 26674543 PMC4682184

[51] Xu Q Thompson JE Carroll M. mTOR Regulates Cell Survival After Etoposide Treatment in Primary AML Cells. Blood (2005) 106(13):4261–8. doi: 10.1182/blood-2004-11-4468 PMC1895255 16150937

[52] Daver N Schlenk RF Russell NH Levis MJ. Targeting FLT3 Mutations in AML: Review of Current Knowledge and Evidence. Leukemia (2019) 33(2):299–312. doi: 10.1038/s41375-018-0357-9 30651634 PMC6365380

[53] Gregory MA D'Alessandro A Alvarez-Calderon F Kim J Nemkov T Adane B. ATM/G6PD-Driven Redox Metabolism Promotes FLT3 Inhibitor Resistance in Acute Myeloid Leukemia. Proc Natl Acad Sci USA (2016) 113(43):E6669–E78. doi: 10.1073/pnas.1603876113 PMC5086999 27791036

[54] Testa U Labbaye C Castelli G Pelosi E. Oxidative Stress and Hypoxia in Normal and Leukemic Stem Cells. Exp Hematol (2016) 44(7):540–60. doi: 10.1016/j.exphem.2016.04.012 27179622

[55] Chen WL Wang YY Zhao A Xia L Xie G Su M. Enhanced Fructose Utilization Mediated by SLC2A5 Is a Unique Metabolic Feature of Acute Myeloid Leukemia With Therapeutic Potential. Cancer Cell (2016) 30(5):779–91. doi: 10.1016/j.ccell.2016.09.006 PMC5496656 27746145

[56] Herst PM Howman RA Neeson PJ Berridge MV Ritchie DS. The Level of Glycolytic Metabolism in Acute Myeloid Leukemia Blasts at Diagnosis is Prognostic for Clinical Outcome. J Leukoc Biol (2011) 89(1):51–5. doi: 10.1189/jlb.0710417 20959411

[57] Spinelli JB Haigis MC. The Multifaceted Contributions of Mitochondria to Cellular Metabolism. Nat Cell Biol (2018) 20(7):745–54. doi: 10.1038/s41556-018-0124-1 PMC6541229 29950572

[58] Oliveira GL Coelho AR Marques R Oliveira PJ. Cancer Cell Metabolism: Rewiring the Mitochondrial Hub. Biochim Biophys Acta Mol Basis Dis (2021) 1867(2):166016. doi: 10.1016/j.bbadis.2020.166016 33246010

[59] Anderson NM Mucka P Kern JG Feng H. The Emerging Role and Targetability of the TCA Cycle in Cancer Metabolism. Protein Cell (2018) 9(2):216–37. doi: 10.1007/s13238-017-0451-1 PMC5818369 28748451

[60] Willems L Jacque N Jacquel A Neveux N Maciel TT Lambert M. Inhibiting Glutamine Uptake Represents an Attractive New Strategy for Treating Acute Myeloid Leukemia. Blood (2013) 122(20):3521–32. doi: 10.1182/blood-2013-03-493163 PMC3829119 24014241

[61] Cluntun AA Lukey MJ Cerione RA Locasale JW. Glutamine Metabolism in Cancer: Understanding the Heterogeneity. Trends Cancer (2017) 3(3):169–80. doi: 10.1016/j.trecan.2017.01.005 PMC5383348 28393116

[62] Lin KH Xie A Rutter JC Ahn YR Lloyd-Cowden JM Nichols AG. Systematic Dissection of the Metabolic-Apoptotic Interface in AML Reveals Heme Biosynthesis to Be a Regulator of Drug Sensitivity. Cell Metab (2019) 29(5):1217–31 e7. doi: 10.1016/j.cmet.2019.01.011 30773463 PMC6506362

[63] Yang C Ko B Hensley CT Jiang L Wasti AT Kim J. Glutamine Oxidation Maintains the TCA Cycle and Cell Survival During Impaired Mitochondrial Pyruvate Transport. Mol Cell (2014) 56(3):414–24. doi: 10.1016/j.molcel.2014.09.025 PMC4268166 25458842

[64] Takubo K Nagamatsu G Kobayashi CI Nakamura-Ishizu A Kobayashi H Ikeda E. Regulation of Glycolysis by Pdk Functions as a Metabolic Checkpoint for Cell Cycle Quiescence in Hematopoietic Stem Cells. Cell Stem Cell (2013) 12(1):49–61. doi: 10.1016/j.stem.2012.10.011 23290136 PMC6592822

[65] Ward PS Patel J Wise DR Abdel-Wahab O Bennett BD Coller HA. The Common Feature of Leukemia-Associated IDH1 and IDH2 Mutations is a Neomorphic Enzyme Activity Converting Alpha-Ketoglutarate to 2-Hydroxyglutarate. Cancer Cell (2010) 17(3):225–34. doi: 10.1016/j.ccr.2010.01.020 PMC2849316 20171147

[66] Kats LM Reschke M Taulli R Pozdnyakova O Burgess K Bhargava P. Proto-Oncogenic Role of Mutant IDH2 in Leukemia Initiation and Maintenance. Cell Stem Cell (2014) 14(3):329–41. doi: 10.1016/j.stem.2013.12.016 PMC4380188 24440599

[67] Jan M Snyder TM Corces-Zimmerman MR Vyas P Weissman IL Quake SR. Clonal Evolution of Preleukemic Hematopoietic Stem Cells Precedes Human Acute Myeloid Leukemia. Sci Transl Med (2012) 4(149):149ra18. doi: 10.1126/scitranslmed.3004315 PMC4045621 22932223

[68] Chou WC Peng KY Lei WC Ko BS Tsay W Kuo CH. Persistence of Mutant Isocitrate Dehydrogenase in Patients With Acute Myeloid Leukemia in Remission. Leukemia (2012) 26(3):527–9. doi: 10.1038/leu.2011.215 21844873

[69] Chou WC Hou HA Chen CY Tang JL Yao M Tsay W. Distinct Clinical and Biologic Characteristics in Adult Acute Myeloid Leukemia Bearing the Isocitrate Dehydrogenase 1 Mutation. Blood (2010) 115(14):2749–54. doi: 10.1182/blood-2009-11-253070 20097881

[70] Shlush LI Zandi S Mitchell A Chen WC Brandwein JM Gupta V. Identification of Pre-Leukaemic Haematopoietic Stem Cells in Acute Leukaemia. Nature (2014) 506(7488):328–33. doi: 10.1038/nature13038 PMC4991939 24522528

[71] Ashton TM McKenna WG Kunz-Schughart LA Higgins GS. Oxidative Phosphorylation as an Emerging Target in Cancer Therapy. Clin Cancer Res (2018) 24(11):2482–90. doi: 10.1158/1078-0432.CCR-17-3070 29420223

[72] Sriskanthadevan S Jeyaraju DV Chung TE Prabha S Xu W Skrtic M. AML Cells Have Low Spare Reserve Capacity in Their Respiratory Chain That Renders Them Susceptible to Oxidative Metabolic Stress. Blood (2015) 125(13):2120–30. doi: 10.1182/blood-2014-08-594408 PMC4375109 25631767

[73] Skrtic M Sriskanthadevan S Jhas B Gebbia M Wang X Wang Z. Inhibition of Mitochondrial Translation as a Therapeutic Strategy for Human Acute Myeloid Leukemia. Cancer Cell (2011) 20(5):674–88. doi: 10.1016/j.ccr.2011.10.015 PMC3221282 22094260

[74] Cole A Wang Z Coyaud E Voisin V Gronda M Jitkova Y. Inhibition of the Mitochondrial Protease ClpP as a Therapeutic Strategy for Human Acute Myeloid Leukemia. Cancer Cell (2015) 27(6):864–76. doi: 10.1016/j.ccell.2015.05.004 PMC4461837 26058080

[75] Lieu EL Nguyen T Rhyne S Kim J. Amino Acids in Cancer. Exp Mol Med (2020) 52(1):15–30. doi: 10.1038/s12276-020-0375-3 31980738 PMC7000687

[76] Gallipoli P Giotopoulos G Tzelepis K Costa ASH Vohra S Medina-Perez P. Glutaminolysis is a Metabolic Dependency in FLT3(ITD) Acute Myeloid Leukemia Unmasked by FLT3 Tyrosine Kinase Inhibition. Blood (2018) 131(15):1639–53. doi: 10.1182/blood-2017-12-820035 PMC6061932 29463564

[77] Cormerais Y Massard PA Vucetic M Giuliano S Tambutte E Durivault J. The Glutamine Transporter ASCT2 (SLC1A5) Promotes Tumor Growth Independently of the Amino Acid Transporter LAT1 (Slc7a5). J Biol Chem (2018) 293(8):2877–87. doi: 10.1074/jbc.RA117.001342 PMC5827425 29326164

[78] Jacque N Ronchetti AM Larrue C Meunier G Birsen R Willems L. Targeting Glutaminolysis has Antileukemic Activity in Acute Myeloid Leukemia and Synergizes With BCL-2 Inhibition. Blood (2015) 126(11):1346–56. doi: 10.1182/blood-2015-01-621870 PMC4608389 26186940

[79] Stevens BM Jones CL Pollyea DA Culp-Hill R D'Alessandro A Winters A. Fatty Acid Metabolism Underlies Venetoclax Resistance in Acute Myeloid Leukemia Stem Cells. Nat Cancer (2020) 1(12):1176–87. doi: 10.1038/s43018-020-00126-z PMC8054994 33884374

[80] Raffel S Falcone M Kneisel N Hansson J Wang W Lutz C. BCAT1 Restricts alphaKG Levels in AML Stem Cells Leading to IDHmut-Like DNA Hypermethylation. Nature (2017) 551(7680):384–8. doi: 10.1038/nature24294 29144447

[81] Miraki-Moud F Ghazaly E Ariza-McNaughton L Hodby KA Clear A Anjos-Afonso F. Arginine Deprivation Using Pegylated Arginine Deiminase has Activity Against Primary Acute Myeloid Leukemia Cells In Vivo. Blood (2015) 125(26):4060–8. doi: 10.1182/blood-2014-10-608133 25896651

[82] Mussai F Wheat R Sarrou E Booth S Stavrou V Fultang L. Targeting the Arginine Metabolic Brake Enhances Immunotherapy for Leukaemia. Int J Cancer (2019) 145(8):2201–8. doi: 10.1002/ijc.32028 PMC6767531 30485425

[83] Raffel S Hansson J Falcone M Lutz C Bischel O Ho AD. Characteristic Amino Acid and Energy Metabolism in AML Stem Cells As Revealed By Quantitative Multiplex Proteomics. Blood (2018) 132(Supplement 1):2780–. doi: 10.1182/blood-2018-99-117046

[84] Maher M Diesch J Casquero R Buschbeck M. Epigenetic-Transcriptional Regulation of Fatty Acid Metabolism and Its Alterations in Leukaemia. Front Genet (2018) 9:405. doi: 10.3389/fgene.2018.00405 30319689 PMC6165860

[85] Snaebjornsson MT Janaki-Raman S Schulze A. Greasing the Wheels of the Cancer Machine: The Role of Lipid Metabolism in Cancer. Cell Metab (2020) 31(1):62–76. doi: 10.1016/j.cmet.2019.11.010 31813823

[86] Pabst T Kortz L Fiedler GM Ceglarek U Idle JR Beyoglu D. The Plasma Lipidome in Acute Myeloid Leukemia at Diagnosis in Relation to Clinical Disease Features. BBA Clin (2017) 7:105–14. doi: 10.1016/j.bbacli.2017.03.002 PMC5357680 28331812

[87] Balko JM Schwarz LJ Luo N Estrada MV Giltnane JM Davila-Gonzalez D. Triple-Negative Breast Cancers With Amplification of JAK2 at the 9p24 Locus Demonstrate JAK2-Specific Dependence. Sci Transl Med (2016) 8(334):334ra53. doi: 10.1126/scitranslmed.aad3001 PMC5256931 27075627

[88] Samudio I Konopleva M. Targeting Leukemia’s “Fatty Tooth”. Blood (2015) 126(16):1874–5. doi: 10.1182/blood-2015-08-665125 26472736

[89] Humbert M Seiler K Mosimann S Rentsch V Sharma K Pandey AV. Reducing FASN Expression Sensitizes Acute Myeloid Leukemia Cells to Differentiation Therapy. Cell Death Differ (2021) 28(8):2465–81. doi: 10.1101/2020.01.29.924555 PMC8329134 33742137

[90] Tabe Y Konopleva M Andreeff M. Fatty Acid Metabolism, Bone Marrow Adipocytes, and AML. Front Oncol (2020) 10:155. doi: 10.3389/fonc.2020.00155 32133293 PMC7040225

[91] Pietrocola F Galluzzi L Bravo-San Pedro JM Madeo F Kroemer G. Acetyl Coenzyme A: A Central Metabolite and Second Messenger. Cell Metab (2015) 21(6):805–21. doi: 10.1016/j.cmet.2015.05.014 26039447

[92] Shi J Fu H Jia Z He K Fu L Wang W. High Expression of CPT1A Predicts Adverse Outcomes: A Potential Therapeutic Target for Acute Myeloid Leukemia. EBioMedicine (2016) 14:55–64. doi: 10.1016/j.ebiom.2016.11.025 27916548 PMC5161445

[93] Wu Y Hurren R MacLean N Gronda M Jitkova Y Sukhai MA. Carnitine Transporter CT2 (SLC22A16) is Over-Expressed in Acute Myeloid Leukemia (AML) and Target Knockdown Reduces Growth and Viability of AML Cells. Apoptosis (2015) 20(8):1099–108. doi: 10.1007/s10495-015-1137-x 25998464

[94] Ito K Carracedo A Weiss D Arai F Ala U Avigan DE. A PML-PPAR-Delta Pathway for Fatty Acid Oxidation Regulates Hematopoietic Stem Cell Maintenance. Nat Med (2012) 18(9):1350–8. doi: 10.1038/nm.2882 PMC3566224 22902876

[95] Loeffler D Schroeder T. Symmetric and Asymmetric Activation of Hematopoietic Stem Cells. Curr Opin Hematol (2021) 28(4):262–8. doi: 10.1097/MOH.0000000000000644 34059600

[96] Shafat MS Oellerich T Mohr S Robinson SD Edwards DR Marlein CR. Leukemic Blasts Program Bone Marrow Adipocytes to Generate a Protumoral Microenvironment. Blood (2017) 129(10):1320–32. doi: 10.1182/blood-2016-08-734798 28049638

[97] Powell JA Wallington-Beddoe CT Pitson SM. Targeting Sphingosine Kinase 1 in Acute Myeloid Leukemia: Translation to Clinic. Int J Hematol Oncol (2017) 6(2):31–4. doi: 10.2217/ijh-2017-0011 PMC6171997 30302220

[98] Ghazaly EA Miraki-Moud F Smith P Gnanaranjan C Koniali L Oke A. Repression of Sphingosine Kinase (SK)-Interacting Protein (SKIP) in Acute Myeloid Leukemia Diminishes SK Activity and its Re-Expression Restores SK Function. J Biol Chem (2020) 295(16):5496–508. doi: 10.1074/jbc.RA119.010467 PMC7170527 32161116

[99] Powell JA Lewis AC Zhu W Toubia J Pitman MR Wallington-Beddoe CT. Targeting Sphingosine Kinase 1 Induces MCL1-Dependent Cell Death in Acute Myeloid Leukemia. Blood (2017) 129(6):771–82. doi: 10.1182/blood-2016-06-720433 PMC7484978 27956387

[100] Xie SZ Kaufmann KB Wang W Chan-Seng-Yue M Gan OI Laurenti E. Sphingosine-1-Phosphate Receptor 3 Potentiates Inflammatory Programs in Normal and Leukemia Stem Cells to Promote Differentiation. Blood Cancer Discov (2021) 2(1):32–53. doi: 10.1158/2643-3230.BCD-20-0155 33458693 PMC7116590

[101] Corces MR Chang HY Majeti R. Preleukemic Hematopoietic Stem Cells in Human Acute Myeloid Leukemia. Front Oncol (2017) 7:263. doi: 10.3389/fonc.2017.00263 29164062 PMC5681525

[102] Shlush LI Mitchell A Heisler L Abelson S Ng SWK Trotman-Grant A. Tracing the Origins of Relapse in Acute Myeloid Leukaemia to Stem Cells. Nature (2017) 547(7661):104–8. doi: 10.1038/nature22993 28658204

[103] Garg M Nagata Y Kanojia D Mayakonda A Yoshida K Haridas Keloth S. Profiling of Somatic Mutations in Acute Myeloid Leukemia With FLT3-ITD at Diagnosis and Relapse. Blood (2015) 126(22):2491–501. doi: 10.1182/blood-2015-05-646240 PMC4661172 26438511

[104] Ding L Ley TJ Larson DE Miller CA Koboldt DC Welch JS. Clonal Evolution in Relapsed Acute Myeloid Leukaemia Revealed by Whole-Genome Sequencing. Nature (2012) 481(7382):506–10. doi: 10.1038/nature10738 PMC3267864 22237025

[105] Vu LP Kharas MG. Targeting the Residual Leukemia Cells After Chemotherapy. Cancer Cell (2018) 34(3):353–5. doi: 10.1016/j.ccell.2018.08.012 30205040

[106] Hira VVV Van Noorden CJF Carraway HE Maciejewski JP Molenaar RJ. Novel Therapeutic Strategies to Target Leukemic Cells That Hijack Compartmentalized Continuous Hematopoietic Stem Cell Niches. Biochim Biophys Acta Rev Cancer (2017) 1868(1):183–98. doi: 10.1016/j.bbcan.2017.03.010 28363872

[107] van Gils N Denkers F Smit L. Escape From Treatment; the Different Faces of Leukemic Stem Cells and Therapy Resistance in Acute Myeloid Leukemia. Front Oncol (2021) 11:659253. doi: 10.3389/fonc.2021.659253 34012921 PMC8126717

[108] Vidal RS Quarti J Rodrigues MF Rumjanek FD Rumjanek VM. Metabolic Reprogramming During Multidrug Resistance in Leukemias. Front Oncol (2018) 8:90. doi: 10.3389/fonc.2018.00090 29675398 PMC5895924

[109] Salvia AM Cuviello F Coluzzi S Nuccorini R Attolico I Pascale SP. Expression of Some ATP-Binding Cassette Transporters in Acute Myeloid Leukemia. Hematol Rep (2017) 9(4):7406–. doi: 10.4081/hr.2017.7406 PMC5757415 29333226

[110] Saito Y Uchida N Tanaka S Suzuki N Tomizawa-Murasawa M Sone A. Induction of Cell Cycle Entry Eliminates Human Leukemia Stem Cells in a Mouse Model of AML. Nat Biotechnol (2010) 28(3):275–80. doi: 10.1038/nbt.1607 PMC3857633 20160717

[111] Farge T Saland E de Toni F Aroua N Hosseini M Perry R. Chemotherapy-Resistant Human Acute Myeloid Leukemia Cells Are Not Enriched for Leukemic Stem Cells But Require Oxidative Metabolism. Cancer Discov (2017) 7(7):716–35. doi: 10.1158/2159-8290.CD-16-0441 PMC5501738 28416471

[112] Larrue C Saland E Vergez F Serhan N Delabesse E Mansat-De Mas V. Antileukemic Activity of 2-Deoxy-D-Glucose Through Inhibition of N-Linked Glycosylation in Acute Myeloid Leukemia With FLT3-ITD or C-KIT Mutations. Mol Cancer Ther (2015) 14(10):2364–73. doi: 10.1158/1535-7163.MCT-15-0163 26206337

[113] Huang A Ju HQ Liu K Zhan G Liu D Wen S. Metabolic Alterations and Drug Sensitivity of Tyrosine Kinase Inhibitor Resistant Leukemia Cells With a FLT3/ITD Mutation. Cancer Lett (2016) 377(2):149–57. doi: 10.1016/j.canlet.2016.04.040 27132990

[114] Sykes DB Kfoury YS Mercier FE Wawer MJ Law JM Haynes MK. Inhibition of Dihydroorotate Dehydrogenase Overcomes Differentiation Blockade in Acute Myeloid Leukemia. Cell (2016) 167(1):171–86.e15. doi: 10.1016/j.cell.2016.08.057 27641501 PMC7360335

[115] Wu D Wang W Chen W Lian F Lang L Huang Y. Pharmacological Inhibition of Dihydroorotate Dehydrogenase Induces Apoptosis and Differentiation in Acute Myeloid Leukemia Cells. Haematologica (2018) 103(9):1472–83. doi: 10.3324/haematol.2018.188185 PMC6119157 29880605

[116] Konopleva M Pollyea DA Potluri J Chyla B Hogdal L Busman T. Efficacy and Biological Correlates of Response in a Phase II Study of Venetoclax Monotherapy in Patients With Acute Myelogenous Leukemia. Cancer Discov (2016) 6(10):1106–17. doi: 10.1158/2159-8290.CD-16-0313 PMC5436271 27520294

[117] Pollyea DA Stevens BM Jones CL Winters A Pei S Minhajuddin M. Venetoclax With Azacitidine Disrupts Energy Metabolism and Targets Leukemia Stem Cells in Patients With Acute Myeloid Leukemia. Nat Med (2018) 24(12):1859–66. doi: 10.1038/s41591-018-0233-1 PMC7001730 30420752

[118] DiNardo CD Pratz KW Letai A Jonas BA Wei AH Thirman M. Safety and Preliminary Efficacy of Venetoclax With Decitabine or Azacitidine in Elderly Patients With Previously Untreated Acute Myeloid Leukaemia: A non-Randomised, Open-Label, Phase 1b Study. Lancet Oncol (2018) 19(2):216–28. doi: 10.1016/S1470-2045(18)30010-X 29339097

[119] Mali RS Zhang Q DeFilippis R Cavazos A Kuruvilla VM Raman J. Venetoclax Combines Synergistically With FLT3 Inhibition to Effectively Target Leukemic Cells in FLT3-ITD+ Acute Myeloid Leukemia Models. Haematologica (2020) 106(4):1034–46. doi: 10.3324/haematol.2019.244020 PMC8017817 32414851

[120] Reed GA Schiller GJ Kambhampati S Tallman MS Douer D Minden MD. A Phase 1 Study of Intravenous Infusions of Tigecycline in Patients With Acute Myeloid Leukemia. Cancer Med (2016) 5(11):3031–40. doi: 10.1002/cam4.845 PMC5119957 27734609

[121] Liyanage SU Hurren R Voisin V Bridon G Wang X Xu C. Leveraging Increased Cytoplasmic Nucleoside Kinase Activity to Target mtDNA and Oxidative Phosphorylation in AML. Blood (2017) 129(19):2657–66. doi: 10.1182/blood-2016-10-741207 PMC5766841 28283480

[122] Stein EM DiNardo CD Pollyea DA Fathi AT Roboz GJ Altman JK. Enasidenib in Mutant IDH2 Relapsed or Refractory Acute Myeloid Leukemia. Blood (2017) 130(6):722–31. doi: 10.1182/blood-2017-04-779405 PMC5572791 28588020

[123] Amatangelo MD Quek L Shih A Stein EM Roshal M David MD. Enasidenib Induces Acute Myeloid Leukemia Cell Differentiation to Promote Clinical Response. Blood (2017) 130(6):732–41. doi: 10.1182/blood-2017-04-779447 PMC5553578 28588019

[124] Yen K Travins J Wang F David MD Artin E Straley K. AG-221, a First-In-Class Therapy Targeting Acute Myeloid Leukemia Harboring Oncogenic IDH2 Mutations. Cancer Discov (2017) 7(5):478–93. doi: 10.1158/2159-8290.CD-16-1034 28193778

[125] Ahmed T Holwerda S Klepin HD Isom S Ellis LR Lyerly S. High Dose Cytarabine, Mitoxantrone and L-Asparaginase (HAMA) Salvage for Relapsed or Refractory Acute Myeloid Leukemia (AML) in the Elderly. Leuk Res (2015) 39(9):945–9. doi: 10.1016/j.leukres.2015.05.010 PMC4546894 26154683

[126] Michelozzi IM Granata V De Ponti G Alberti G Tomasoni C Antolini L. Acute Myeloid Leukaemia Niche Regulates Response to L-Asparaginase. Br J Haematol (2019) 186(3):420–30. doi: 10.1111/bjh.15920 31044436

[127] Thomas XG Tardy ET Guieze R Chevallier P Marolleau JP Orsini F. GRASPA-AML 2012-01 Study (NCT01810705): A Multicenter, Open, Randomized Phase 2b Trial Evaluating ERY001 (L-Asparaginase Encapsulated in Red Blood Cells) Plus Low-Dose Cytarabine vs Low-Dose Cytarabine Alone, in Treatment of Newly Diagnosed Acute Myeloid Leukemia (AML) Elderly Patients, Unfit for Intensive Chemotherapy. Am J Clin Oncol (2015) 33(15_suppl):TPS7099–TPS. doi: 10.1200/jco.2015.33.15_suppl.tps7099

[128] Wang ES Frankfurt O Orford KW Bennett M Flinn IW Maris M. Phase 1 Study of CB-839, a First-In-Class, Orally Administered Small Molecule Inhibitor of Glutaminase in Patients With Relapsed/Refractory Leukemia. Blood (2015) 126(23):2566–. doi: 10.1182/blood.V126.23.2566.2566

[129] Lee EA Angka L Rota SG Hanlon T Mitchell A Hurren R. Targeting Mitochondria With Avocatin B Induces Selective Leukemia Cell Death. Cancer Res (2015) 75(12):2478–88. doi: 10.1158/0008-5472.CAN-14-2676 26077472

[130] Pardee T DeFord-Watts LM Peronto E Levitan DA Hurd DD Kridel S. Altered Lipid and Mitochondrial Metabolism Are Viable Targets in Acute Leukemia. Blood (2011) 118(21):3618–. doi: 10.1182/blood.V118.21.3618.3618

[131] Estan MC Calvino E Calvo S Guillen-Guio B Boyano-Adanez Mdel C de Blas E. Apoptotic Efficacy of Etomoxir in Human Acute Myeloid Leukemia Cells. Cooperation With Arsenic Trioxide and Glycolytic Inhibitors, and Regulation by Oxidative Stress and Protein Kinase Activities. PloS One (2014) 9(12):e115250. doi: 10.1371/journal.pone.0115250 25506699 PMC4266683

[132] Tan SF Liu X Fox TE Barth BM Sharma A Turner SD. Acid Ceramidase is Upregulated in AML and Represents a Novel Therapeutic Target. Oncotarget (2016) 7(50):83208–22. doi: 10.18632/oncotarget.13079 PMC5347763 27825124

[133] Lee JS Roberts A Juarez D Vo TT Bhatt S Herzog LO. Statins Enhance Efficacy of Venetoclax in Blood Cancers. Sci Transl Med (2018) 10(445):1240. doi: 10.1126/scitranslmed.aaq1240 PMC6336198 29899021

[134] Ricciardi MR Mirabilii S Allegretti M Licchetta R Calarco A Torrisi MR. Targeting the Leukemia Cell Metabolism by the CPT1a Inhibition: Functional Preclinical Effects in Leukemias. Blood (2015) 126(16):1925–9. doi: 10.1182/blood-2014-12-617498 26276667

[135] Giammarioli AM Gambardella L Barbati C Pietraforte D Tinari A Alberton M. Differential Effects of the Glycolysis Inhibitor 2-Deoxy-D-Glucose on the Activity of Pro-Apoptotic Agents in Metastatic Melanoma Cells, and Induction of a Cytoprotective Autophagic Response. Int J Cancer (2012) 131(4):E337–47. doi: 10.1002/ijc.26420 21913183

[136] Yang H Tabe Y Sekihara K Saito K Ma H Ruvolo V. Novel Oxidative Phosphorylation Inhibitor IACS-010759 Induces AMPK-Dependent Apoptosis of AML Cells. Blood (2017) 130(Supplement 1):1245–. doi: 10.1182/blood.V130.Suppl_1.1245.1245

[137] Sykes DB. The Emergence of Dihydroorotate Dehydrogenase (DHODH) as a Therapeutic Target in Acute Myeloid Leukemia. Expert Opin Ther Targets (2018) 22(11):893–8. doi: 10.1080/14728222.2018.1536748 PMC6457988 30318938

[138] Stacpoole PW. Therapeutic Targeting of the Pyruvate Dehydrogenase Complex/Pyruvate Dehydrogenase Kinase (PDC/PDK) Axis in Cancer. J Natl Cancer Inst (2017) 109(11). doi: 10.1093/jnci/djx071 29059435

[139] Emadi A Carter-Cooper B Sadowska M Wonodi O Lapidus RG Levis M. Synergistic Antileukemic Effect Of Sequential Administration Of Dichloroacetate (DCA) Combined With Arsenic Trioxide (ATO) In Primary Blasts From Patients With Acute Myeloid Leukemia (AML) and FLT3-ITD AML Cell Lines. Blood (2013) 122(21):3955–. doi: 10.1182/blood.V122.21.3955.3955

[140] Khan AUH Rathore MG Allende-Vega N Vo DN Belkhala S Orecchioni S. Human Leukemic Cells Performing Oxidative Phosphorylation (OXPHOS) Generate an Antioxidant Response Independently of Reactive Oxygen Species (ROS) Production. EBioMedicine (2016) 3:43–53. doi: 10.1016/j.ebiom.2015.11.045 26870816 PMC4739420

[141] DiNardo CD Jonas BA Pullarkat V Thirman MJ Garcia JS Wei AH. Azacitidine and Venetoclax in Previously Untreated Acute Myeloid Leukemia. N Engl J Med (2020) 383(7):617–29. doi: 10.1056/NEJMoa2012971 32786187

[142] Huemer F Melchardt T Jansko B Wahida A Jilg S Jost PJ. Durable Remissions With Venetoclax Monotherapy in Secondary AML Refractory to Hypomethylating Agents and High Expression of BCL-2 and/or BIM. Eur J Haematol (2019) 102(5):437–41. doi: 10.1111/ejh.13218 PMC6849823 30725494

[143] Samudio I Harmancey R Fiegl M Kantarjian H Konopleva M Korchin B. Pharmacologic Inhibition of Fatty Acid Oxidation Sensitizes Human Leukemia Cells to Apoptosis Induction. J Clin Invest (2010) 120(1):142–56. doi: 10.1172/JCI38942 PMC2799198 20038799

[144] Matre P Velez J Jacamo R Qi Y Su X Cai T. Inhibiting Glutaminase in Acute Myeloid Leukemia: Metabolic Dependency of Selected AML Subtypes. Oncotarget (2016) 7(48):79722–35. doi: 10.18632/oncotarget.12944 PMC5340236 27806325

[145] Gregory MA Nemkov T Park HJ Zaberezhnyy V Gehrke S Adane B. Targeting Glutamine Metabolism and Redox State for Leukemia Therapy. Clin Cancer Res (2019) 25(13):4079–90. doi: 10.1158/1078-0432.CCR-18-3223 PMC6642698 30940653

[146] Gregory MA Nemkov T Reisz JA Zaberezhnyy V Hansen KC D'Alessandro A. Glutaminase Inhibition Improves FLT3 Inhibitor Therapy for Acute Myeloid Leukemia. Exp Hematol (2018) 58:52–8. doi: 10.1016/j.exphem.2017.09.007 PMC5815916 28947392

[147] Jones CL Stevens BM Pollyea DA Culp-Hill R Reisz JA Nemkov T. Nicotinamide Metabolism Mediates Resistance to Venetoclax in Relapsed Acute Myeloid Leukemia Stem Cells. Cell Stem Cell (2020) 27(5):748–64.e4. doi: 10.1016/j.stem.2020.07.021 32822582 PMC7655603

[148] Jones CL Stevens BM D'Alessandro A Culp-Hill R Reisz JA Pei S. Cysteine Depletion Targets Leukemia Stem Cells Through Inhibition of Electron Transport Complex II. Blood (2019) 134(4):389–94. doi: 10.1182/blood.2019898114 PMC6659257 31101624

[149] Luo M Brooks M Wicha MS. Asparagine and Glutamine: Co-Conspirators Fueling Metastasis. Cell Metab (2018) 27(5):947–9. doi: 10.1016/j.cmet.2018.04.012 29719230

[150] Patzke CL Duffy AP Duong VH El Chaer F Trovato JA Baer MR. Comparison of High-Dose Cytarabine, Mitoxantrone, and Pegaspargase (HAM-Pega) to High-Dose Cytarabine, Mitoxantrone, Cladribine, and Filgrastim (CLAG-M) as First-Line Salvage Cytotoxic Chemotherapy for Relapsed/Refractory Acute Myeloid Leukemia. J Clin Med (2020) 9(2):536. doi: 10.3390/jcm9020536 PMC7074083 32079074

[151] Mussai F Egan S Higginbotham-Jones J Perry T Beggs A Odintsova E. Arginine Dependence of Acute Myeloid Leukemia Blast Proliferation: A Novel Therapeutic Target. Blood (2015) 125(15):2386–96. doi: 10.1182/blood-2014-09-600643 PMC4416943 25710880

[152] Stuani L Riols F Millard P Sabatier M Batut A Saland E. Stable Isotope Labeling Highlights Enhanced Fatty Acid and Lipid Metabolism in Human Acute Myeloid Leukemia. Int J Mol Sci (2018) 19(11):3325. doi: 10.3390/ijms19113325 PMC6274868 30366412

[153] Dany M Gencer S Nganga R Thomas RJ Oleinik N Baron KD. Targeting FLT3-ITD Signaling Mediates Ceramide-Dependent Mitophagy and Attenuates Drug Resistance in AML. Blood (2016) 128(15):1944–58. doi: 10.1182/blood-2016-04-708750 PMC5064718 27540013

[154] Leni Z Parakkal G Arcaro A. Emerging Metabolic Targets in the Therapy of Hematological Malignancies. BioMed Res Int (2013) 2013:946206. doi: 10.1155/2013/946206 24024216 PMC3759275

[155] Ghaffari P Mardinoglu A Nielsen J. Cancer Metabolism: A Modeling Perspective. Front Physiol (2015) 6:382. doi: 10.3389/fphys.2015.00382 26733270 PMC4679931

[156] Cantor JR Abu-Remaileh M Kanarek N Freinkman E Gao X Louissaint A Jr. Physiologic Medium Rewires Cellular Metabolism and Reveals Uric Acid as an Endogenous Inhibitor of UMP Synthase. Cell (2017) 169(2):258–72.e17. doi: 10.1016/j.cell.2017.03.023 28388410 PMC5421364

[157] Vande Voorde J Ackermann T Pfetzer N Sumpton D Mackay G Kalna G. Improving the Metabolic Fidelity of Cancer Models With a Physiological Cell Culture Medium. Sci Adv (2019) 5(1):eaau7314. doi: 10.1126/sciadv.aau7314 30613774 PMC6314821

[158] Tabe Y Saitoh K Yang H Sekihara K Yamatani K Ruvolo V. Inhibition of FAO in AML Co-Cultured With BM Adipocytes: Mechanisms of Survival and Chemosensitization to Cytarabine. Sci Rep (2018) 8(1):16837. doi: 10.1038/s41598-018-35198-6 30442990 PMC6237992

[159] Horibata S Gui G Lack J DeStefano CB Gottesman MM Hourigan CS. Heterogeneity in Refractory Acute Myeloid Leukemia. Proc Natl Acad Sci USA (2019) 116(21):10494–503. doi: 10.1073/pnas.1902375116 PMC6535032 31064876

